# Human pegivirus alters brain and blood immune and transcriptomic profiles of patients with Parkinson’s disease

**DOI:** 10.1172/jci.insight.189988

**Published:** 2025-07-08

**Authors:** Barbara A. Hanson, Xin Dang, Pouya Jamshidi, Alicia Steffens, Kaleigh Copenhaver, Zachary S. Orban, Bernabe Bustos, Steven J. Lubbe, Rudolph J. Castellani, Igor J. Koralnik

**Affiliations:** 1 Davee Department of Neurology,; 2 Department of Pathology,; 3 Department of Neurological Surgery, and; 4 Simpson Querrey Center for Neurogenetics, Northwestern University Feinberg School of Medicine, Chicago, Illinois, USA.

**Keywords:** Neuroscience, Virology, Genetic variation, Neurodegeneration, Parkinson disease

## Abstract

Parkinson’s disease (PD) is a neurodegenerative disorder with both genetic and environmental factors contributing to pathogenesis. Viral infections are potential environmental triggers that influence PD pathology. Using ViroFind, an unbiased platform for whole virome sequencing, along with quantitative PCR (qPCR), we identified human pegivirus (HPgV) in 5 of 10 (50%) of PD brains, confirmed by IHC in 2 of 2 cases, suggesting an association with PD. All 14 age- and sex-matched controls were HPgV negative. HPgV-brain positive patients with PD showed increased neuropathology by Braak stage and Complexin-2 levels, while those positive in the blood had higher IGF-1 and lower pS65-ubiquitin, supporting disruption in metabolism or mitophagy in response to HPgV. RNA-Seq revealed altered immune signaling in HPgV-infected PD samples, including consistent suppression of IL-4 signaling in both the brain and blood. Longitudinal analysis of blood samples showed a genotype-dependent viral response, with HPgV titers correlating directly with IL-4 signaling in a *LRRK2* genotype–dependent manner. *YWHAB* was a key hub gene in the *LRRK2* genotypic response, which exhibited an altered relationship with immune-related factors, including *NFKB1*, *ITPR2*, and *LRRK2* itself, in patients with PD who are positive for HPgV. These results suggest a role for HPgV in shaping PD pathology and highlight the complex interplay between viral infection, immunity, and neuropathogenesis.

## Abstract 2

Human Pegivirus infection may influence Parkinson’s disease by altering immune signaling, metabolism, and key genes, potentially affecting LRRK2 and non-LRRK2 carrier patients differently.

## Graphical abstract

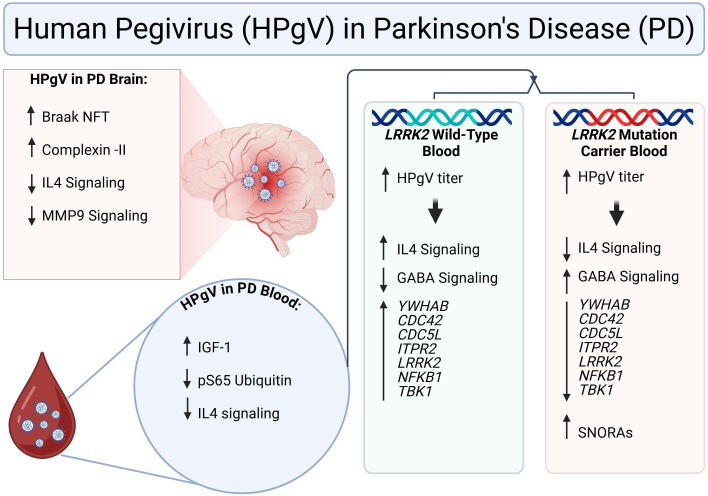

## Introduction

Parkinson’s disease (PD) is a neurodegenerative brain disease affecting 1 million people in the United States, including almost 2% of those older than 65 years (1, 2). It is estimated that more than 10 million people worldwide are living with PD. Genetic associations for PD account for only a small proportion of patients with PD, and it has been hypothesized that PD risk variants interact with environmental factors to cause disease onset (3). Viral infections represent a substantial environmental risk factor, and a number of viruses have been considered as potential causal factors or triggers in the pathogenesis of PD, ever since the occurrence the epidemic of *encephalitis lethargica* (EL) in 1918 (4–16). Indeed, while some patients presented with motor symptoms of Parkinsonism during the acute phase of EL, others experienced later onset after encephalitic Parkinsonism (17, 18).

Parkinsonism has also been observed following Flaviviral encephalitis caused by West Nile virus (WNV), St. Louis Encephalitis virus (SLEV), and Japanese Encephalitis B virus (JEBV), in which MRI findings and therapeutic response to L-dopamine both suggest that the Flaviviral Parkinsonism is caused by a pathology similar to PD (19–22). Patients positive for HIV who develop neurocognitive symptoms may also develop motor symptoms associated with Parkinsonism that are worsened by the progression to AIDS and improved by antiretroviral therapy, though it has been suggested that the Parkinsonism in patients positive for HIV may relate to loss of immune function rather than direct viral involvement (19). These previous studies focused on single viral species, and although abundant data exists, it remains inconclusive to this day what role viral infections play in PD (23).

Metagenomic sequencing has been used to identify viral sequences. However, enrichment of viral targets is necessary prior to sequencing, due to the enormous imbalance between the size and amount of host and viral nucleic acids in human biosamples. To address this issue, we have developed an unbiased metagenomic sequencing–based assay called *ViroFind* that allows us to detect every virus known to infect humans — the entire virome — in clinical biosamples. In a pilot study, *ViroFind* could enrich viral sequences up to 127-fold from human brain samples compared with sequencing alone (24). This assay also allowed us to identify viral sequences in heart biopsies of patients with myocarditis (25) and characterize the virome in brain samples from individuals with HIV infection and substance use disorder (26).

The current study was designed to characterize the entire virome in PD brains and potential mechanisms associated with neurodegeneration. We addressed these questions using *ViroFind* in a unique group of fresh-frozen postmortem brain samples from patients with PD and age- and sex-matched unaffected controls (CT). We then used quantitative PCR (qPCR) to confirm the unique presence of human pegivirus (HPgV) in PD samples and IHC to verify expression of HPgV protein in formalin-fixed, paraffin-embedded (FFPE) contralateral hemisphere. In addition, we performed bulk RNA-Seq analyses to determine differentially expressed genes associated with HPgV infection in the brain. Finally, we used the Parkinson’s Progression Marker Initiative (PPMI) blood samples database to identify HPgV viremia in patients with idiopathic or genetic PD and CTs, and identify transcriptomic alterations associated with HPgV infection in the periphery.

## Results

### Viromic analyses reveal the unique presence of HPgV in PD brain samples.

**Figure 1:**
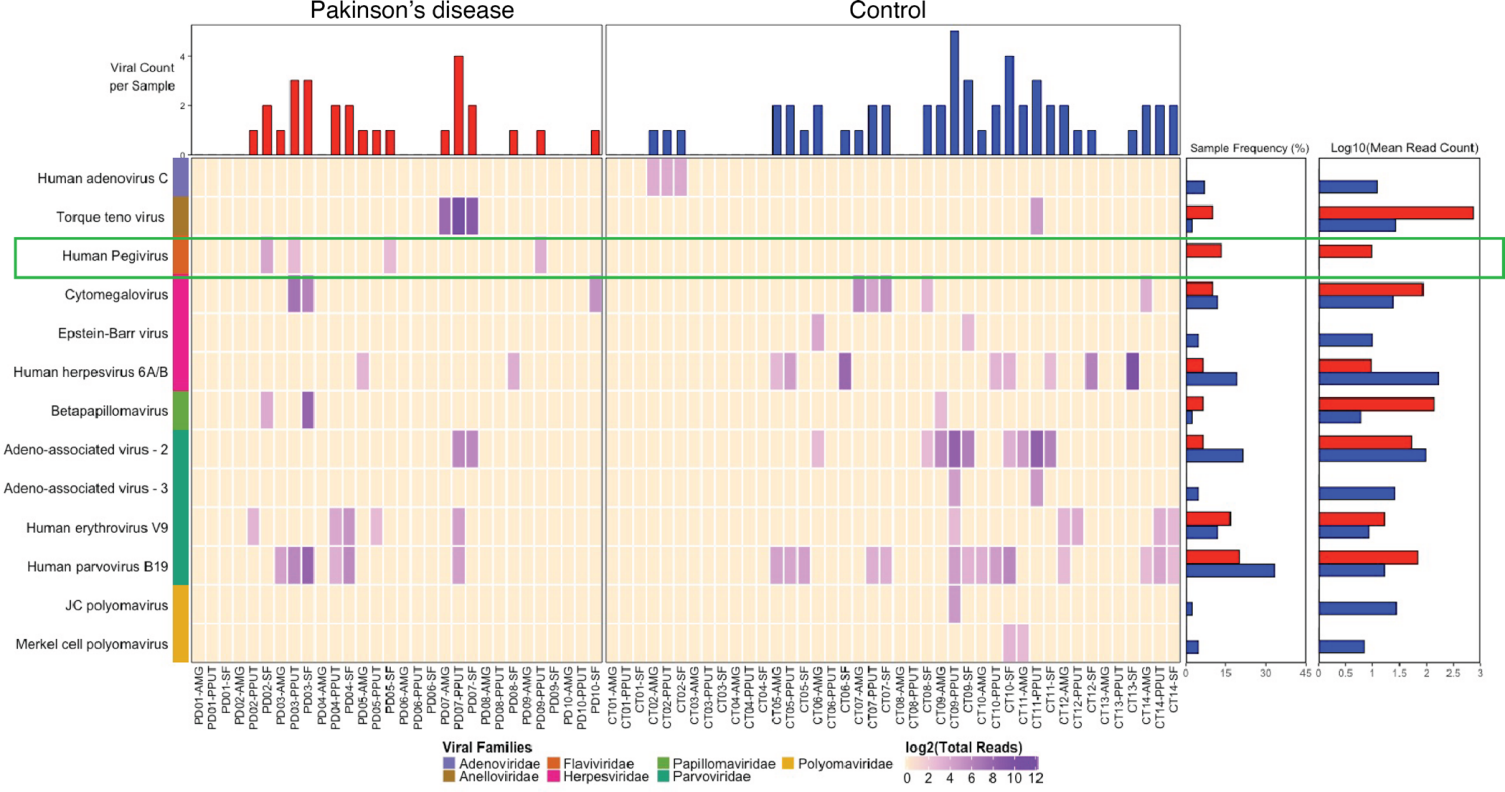
Viruses identified in PD and CT brain tissue by ViroFind. Computed heatmap showing all viral taxa identified by ViroFind library preparation and pipeline with purple log_2_ gradient scale indicating the number of raw viral reads. The frequency of each viral species from amygdala (AMG), posterior putamen (PPUT), and superior frontal cortex (SFC) from 10 individuals with PD (red) and 14 CT individuals (blue) as well as the raw mean read count on a log scale for both groups are shown. Human pegivirus, present only in individuals with PD, is outlined in green.

We analyzed fresh-frozen postmortem brain samples from the amygdala (AMG), posterior putamen (PPUT), and superior frontal cortex (SFC) obtained from 10 patients with PD and 14 age- and sex-matched non-PD CT, provided by the Rush Alzheimer Research Center (RADC) in Chicago, Illinois, USA. Patients with PD were diagnosed clinically according to Core Assessment Program for Intracerebral Transplantation (CAPIT) criteria, as previously described (27). Subject demographics and pathology findings are summarized in Table 1. Brain tissue was analyzed by ViroFind whole virome sequencing and the ViroFind informatics pipeline. Viral taxa identified are displayed in Figure 1. Viruses from 7 viral families were identified. Each patient had 3 brain regions tested for viral presence using whole virome sequencing. Table 2 shows the virome findings for each patient by aggregating viral species identified across the 3 regions. On average, patients with PD had 1.90 ± 1.52 unique viruses across their 3 brain regions, which was not significantly different from the 2.36 ± 2.02 viruses found in CT (*P* = 0.721, Kruskall-Walis). HPgV sequences were identified in 40% (4 of 10) of patients with PD from postmortem brains and 0 of 14 CT brain samples (*P* = 0.020, 2-tailed Fisher’s exact test; Table 2).

### qPCR analysis confirms and expands HPgV detection in PD brains and cerebrospinal fluid.

HPgV presence was confirmed by qPCR targeting the untranslated region (UTR) and nonstructural 3 (*NS3*) gene. qPCR analysis identified an additional patient with PD containing HPgV nucleic acid in the brain tissue. Of 12 CT, who had sufficient brain tissue and matched CSF remaining for qPCR analysis, all samples were confirmed negative (Table 3). Additional qPCR testing of matched cerebrospinal fluid (CSF) and plasma showed that 3 patients with PD and 0 CT had HPgV RNA in the CSF (Table 3). All patients with PD who had HPgV^+^ CSF were previously identified as HPgV^+^ in brain tissue by both ViroFind and qPCR. No patients with PD or CT had HPgV viremia as determined by qPCR of plasma.

### IHC detection of HPgV NS5A protein in brain tissue.

**Figure 2:**
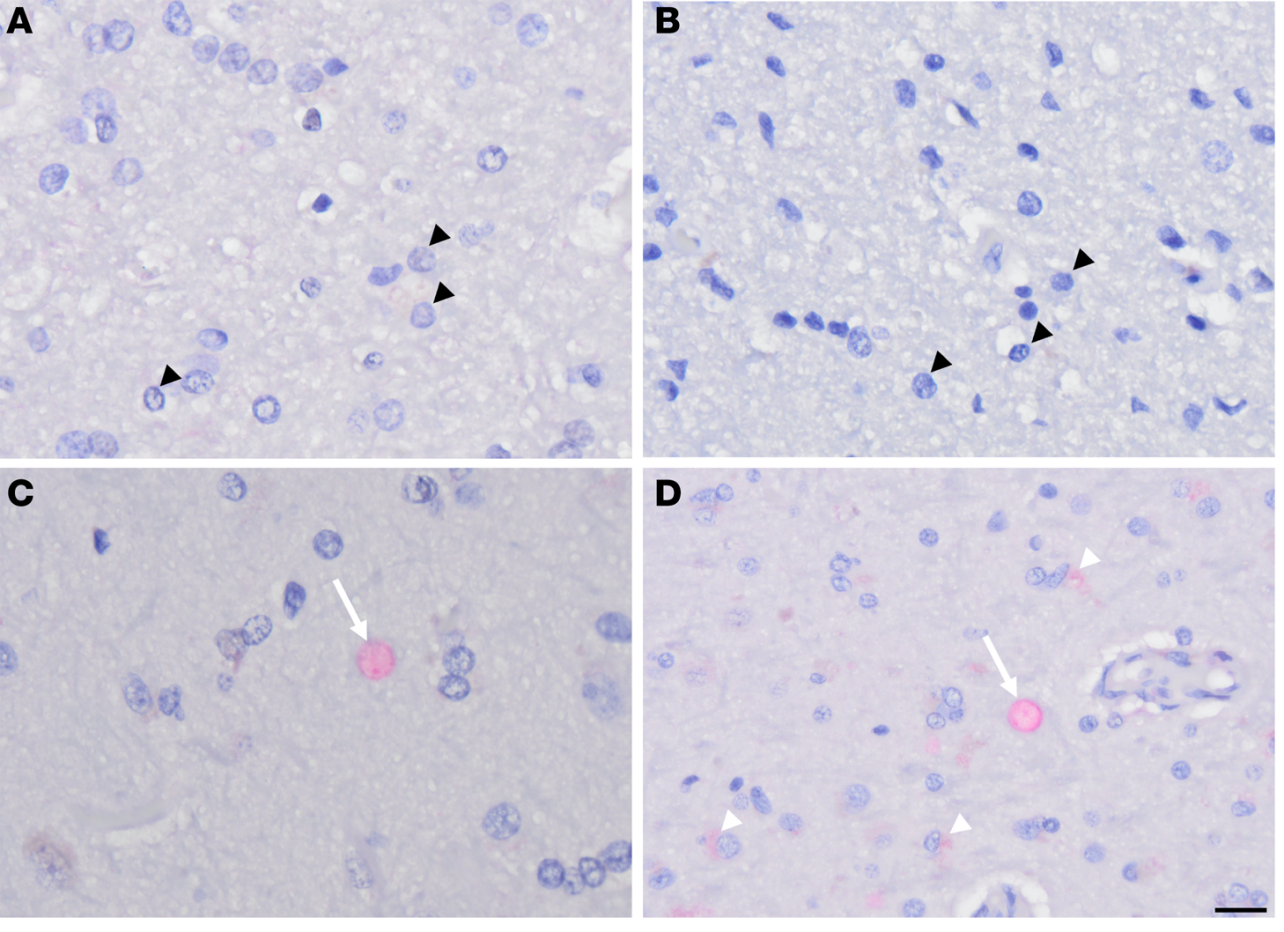
Representative IHC staining of HPgV in control and PD. (**A** and **B**) Subcortical oligodendrocytes (black arrowheads) of 2 control individuals with no evidence of HPgV infection. (**C**) HPgV nuclear immunoreactivity in an oligodendrocyte (white arrow) within the subcortical white matter in a patient with PD. (**D**) Cytoplasmic HPgV immunostaining (white arrowhead) and nuclear HPgV immunoreactivity of an oligodendrocyte (white arrow) within the subcortical white matter in another patient with PD. Scale bar: 20 μm. HPgV, human pegivirus; PD, Parkinson’s disease.

Superior frontal cortical FFPE sections from the hemisphere contralateral to the one used for ViroFind experiments were immunostained for the presence of HPgV nonstructural (NS) protein 5A. Figure 2 shows subcortical white matter from 2 CT individuals (Figure 2, A and B) and 2 patients with PD who were found to be HPgV^+^ with ViroFind (Figure 2, C and D). Oligodendrocytes in CT individuals (Figure 2, A and B) lack HPgV NS5A immunoreactivity while representative oligodendrocytes in PD brains display focal nuclear HPgV NS5A immunoreactivity (Figure 2, C and D) and in the cytoplasm (Figure 2D). These findings suggest that HPgV NS5A protein is present in PD brain tissue, with distinct subcellular localization in oligodendrocytes.

### HPgV in PD brain tissue is associated with limbic braak staging.

Given the difference in HPgV prevalence in PD brains and CSF samples as compared with matched CT, we explored demographic, clinical, or pathological correlations with HPgV positivity (Table 4). HPgV brain tissue–positive patients with PD were of similar age and sex and had similar educational backgrounds as compared with HPgV^–^ patients with PD. Mini Mental State Examination (MMSE) and clinical cognitive diagnoses were similar and in the normal range between HPgV^+^ and HPgV^–^ patients with PD at the time of the last assessment; all patients had MMSE values that indicated no or mild cognitive impairment, with no difference between HPgV^+^ and HPgV^–^ patients (*P* = 0.222, 2-tailed Fisher’s exact test). Braak staging of tau tangles at autopsy indicated that 5 of 5 (100%) patients positive for HPgV (HPgV^+^ patients) with PD had more advanced staging of neurofibrillary tangles (NFT) affecting the limbic region whereas 4 of 5 (80%) HPgV^–^ patients with PD had NFT restricted to the entorhinal cortex (*P* = 0.012, 2-tailed Fisher’s exact test). Other pathological measures, including PD pathology measures of TDP-43 and Lewy Body Disease staging, were similar between patients with PD with and without HPgV. Excitatory Complexin-2, which is responsible for glutamate release, was found to be more densely stained across 6 sampled cortical brain regions in HPgV^+^ compared with HPgV^–^ patients with PD (HPgV^+^, 0.67 [log_10_]; HPgV^–^, –0.34 [log_10_]; *P* = 0.008, 2-tailed *t* test).

### Characterization of the virome in whole blood transcriptomes from the PPMI.

To further investigate these findings, we obtained baseline whole blood transcriptome sequences from 1,393 PPMI participants who were classified as either patients with PD or non-PD CTs in addition to patients who are prodromal with PD and patients who have scans without evidence of dopaminergic deficit (SWEDD). Patients were selected to be age- and sex-matched between groups (Table 5). Raw transcriptome data were processed through the ViroFind bioinformatics pipeline. Viruses identified are summarized in Table 5. Significant differences between PD, prodromal, SWEDD, and CT groups were found for adenovirus C (AdvC), driven by a lower frequency in SWEDD (25.9%) compared with other groups for EBV, driven by a higher burden in CT compared with other groups. HPgV was identified in a total of 14 patients (1.0%) with no significant difference between groups (PD, 8 of 753 [1.1%]; prodromal, 1of 287 [0.3%]; SWEDD, 2 of 54 [3.7%]; CT, 3 of 299 [1.0%]). HPgV viral burden was high across all groups relative to other viruses identified. We further analyzed demographics and genetic variants associated with viral positivity in whole blood of PPMI patients (Supplemental Table 1; supplemental material available online with this article; https://doi.org/10.1172/jci.insight.189988DS1). HPgV positivity was not associated with age, sex, or identified PD variants in *GBA*, *LRRK2*, and *SNCA*.

### HPgV associations with biomarkers in whole blood of patients with PD.

PPMI samples with quantified biomarkers were analyzed based on HPgV positivity in patients with PD (Table 6). Serum levels of Insulin-like growth factor 1 (IGF-1) were significantly higher in HPgV^+^ compared with HPgV^–^ patients with PD (177.85 ng/mL [159.3–190.45] versus 138.40 ng/mL [106.4–160.6]; *P* = 0.047). Conversely, the mitophagy marker phosphorylated ubiquitin at serine 65 (pS65-Ub) was determined to be higher in HPgV^–^ compared with HPgV^+^ patients with PD (46.73 ng/mL [39.13–56.89] versus 32.50 ng/mL [28.44–39.66]; *P* = 0.005). Patients with PD had similar clinical presentation regardless of HPgV positivity in whole blood (Supplemental Table 2). Similar comparison for non-PD groups could not be performed due to low numbers.

### Sequence analysis of PD and CNS associated HPgV strains.

Assembled sequences from all HPgV^+^ samples obtained from brain tissue and CSF from patients with PD, and from whole blood from PD, prodromal PD, SWEDD, and CT, were translated to amino acid (AA) sequence and aligned to HPgV reference sequence NC_001710.1 and an HPgV sequence from the brain parenchyma of a case of HPgV leukoencephalopathy (28) (LE-1: MH179063.1; Supplemental Figure 1) (https://www.ncbi.nlm.nih.gov/nuccore/MH179063.1/). No AA variants were unique among patients with PD nor among sequences from the CNS (brain or CSF). Our analysis confirmed variability in the annotated start codon position for the polyprotein across reference sequences. Experimental data from Simons et al. (29). support translation initiation at methionine (nt 552), leading to the production of the canonical N-terminal sequence encoded by the polyprotein. However, alternative start codons and upstream open reading frames (ORFs) have been identified in other studies. For example, Balcom et al. (28) identified an upstream ORF initiating at an AUG codon prior to the region proposed by Simons et al. (29). Furthermore, our alignment data reveal a premature stop codon shortly after the annotated start site in some isolates (35%; 3 whole blood PD, 1 CSF PD, and 1 whole blood CT), suggesting potential sequence heterogeneity that could affect translation and protein expression. LE-1 was 1 of 2 HPgV sequences, which was previously isolated and published from brain tissue, both of which encode a 32 AA deletion in the NS2 protein. Coverage was obtained from 2 of our CSF isolates for this region, indicating that the deletion is not required for entry into the CNS, at least in the context of PD. Sequences comparisons at the nucleotide level for HPgV genotyping indicated that sequences obtained from all patients and sample types were genotype 2a and 2b (Supplemental Figure 2).

### Transcriptomic effects of HPgV infection in the CNS of patients with PD.

RNA-Seq analysis was performed on the previously analyzed brain tissue samples to determine transcriptomic effects HPgV infection on the CNS of patients with PD. Four PD and 4 CT samples without HPgV infection as well as 3 PD samples with HPgV infection were available for analysis. Poly-A selected RNA was sequenced and differential expression (differentially expressed genes [DEG]) analysis was performed using the Kruskal-Wallis test to accommodate the small number of samples. No genes were found to surpass the Benjamini-Hochberg–adjusted (BH-adjusted) *P* threshold for multiple comparisons, likely due to small groups.

**Figure 3:**
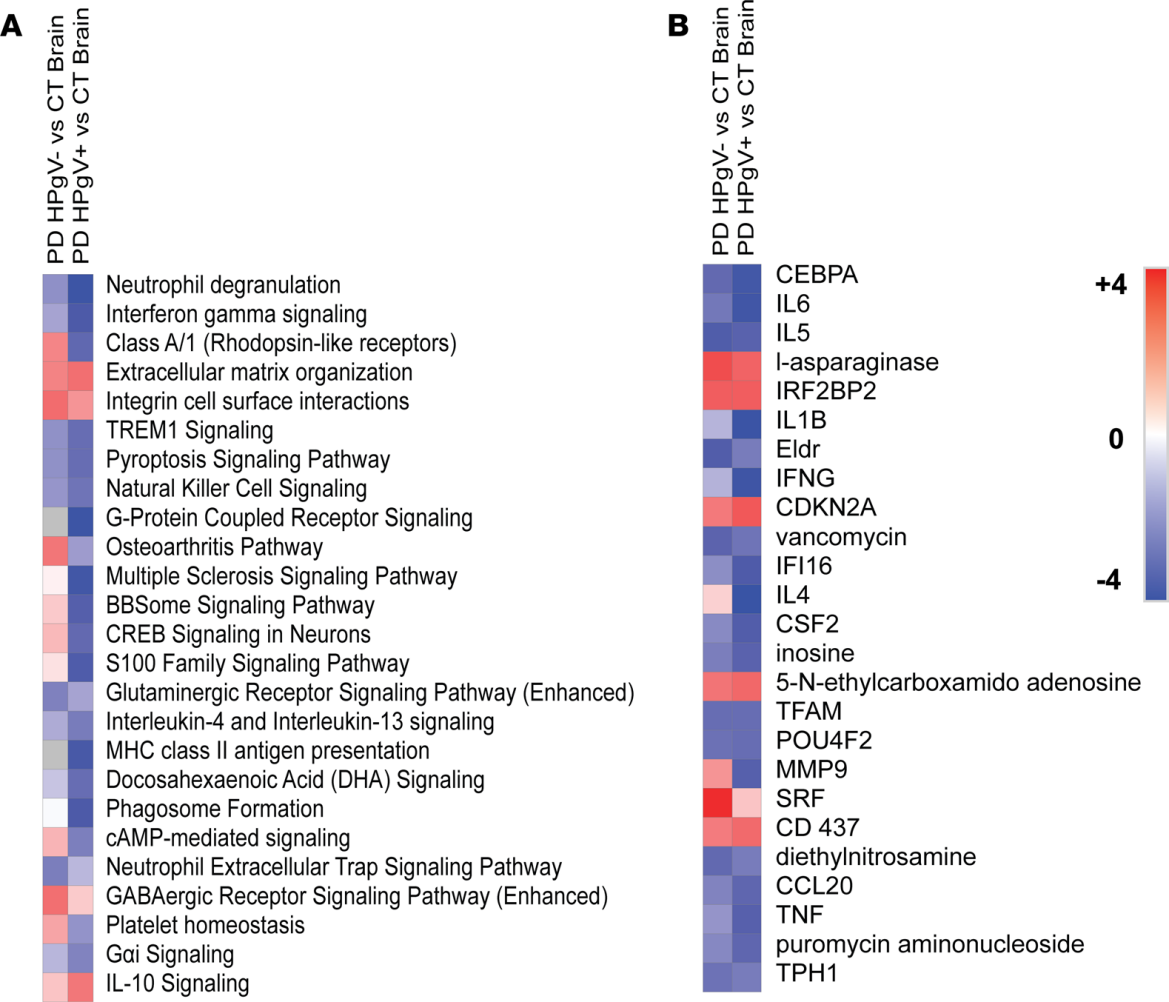
Transcriptomic alterations in HPgV-infected PD brains. (**A** and **B**) Top 25 pathways (**A**) and upstream regulators (**B**), which show enhancement (positive *z* scores, red) or suppression (negative *z* scores, blue), in HPgV^–^ PD brains as compared with CT (left column) or in HPgV^+^ PD brains as compared with CT (right column). Gray boxes indicate no significant pathway/regulator change between conditions. Ranked in order of absolute value, from lowest cumulative rank score to highest. Italicized pathways/regulators indicate an enhancement of the PD phenotype as determined by a more extreme *z* score in the HPgV^+^ as compared with HPgV^–^ analysis.

The top 25 pathways identified as being altered by PD alone (PD HPgV^–^ vs. CT brain) and by PD and HPgV infection (PD HPgV^+^ vs. CT brain) through gene set enrichment analysis (GSEA) are shown in Figure 3A. Upstream regulators were identified based on differential expression of their downstream target genes. The top 25 regulators identified by PD alone (PD HPgV^–^ vs. CT brain) and by PD and HPgV infection (PD HPgV^+^ vs. CT brain) are mapped in Figure 3B.

### Transcriptomic effects of HPgV infection in the whole blood of patients with PD.

HPgV^+^ PD samples were age, sex, and genetic background matched with 3 HPgV^–^ PD and 3 HPgV^–^ CT. Analysis to identify DEGs was performed on variance-stabilized transcripts using 1-way ANOVA (degrees of freedom: between groups = 2; within groups = 8). Two genes showed significantly different expression surpassing the BH *P* adjustment threshold when comparing PD HPgV^–^ patients with PD HPgV^+^ and CT groups.

**Figure 4:**
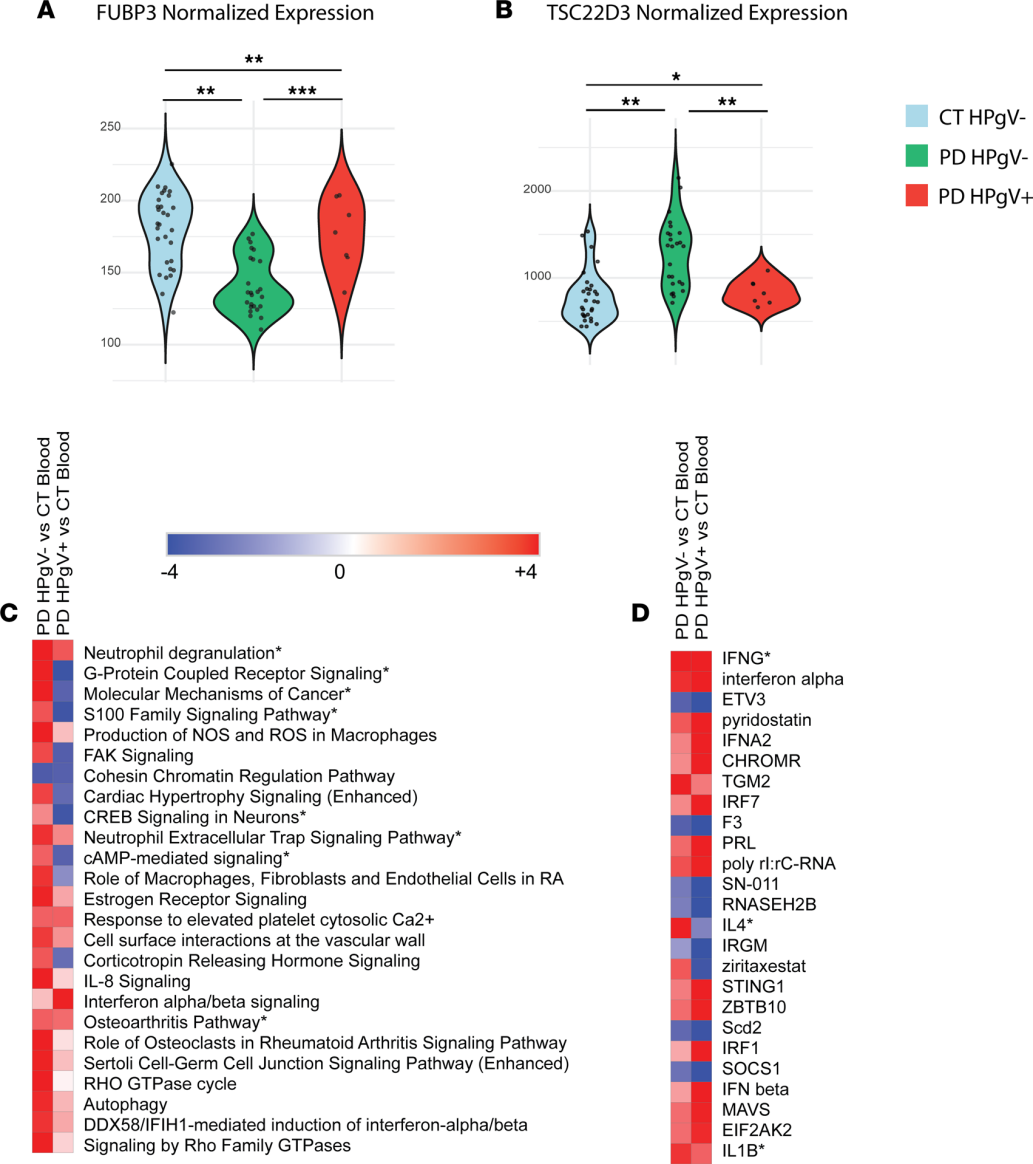
Transcriptomic alterations in HPgV-infected PD whole blood. (**A** and **B**) Violin plots of DEGs by ANOVA (*FUBP3* *P*_adj_ = 0.005; *TSC22D3* *P*_adj_ = 0.012) in whole blood transcriptomes of patients with PD without and with HPgV infection when compared with matched controls. (*P = 0.05-0.01; **P = 0.009-0.001; ***P < 0.001). (**C** and **D**) Top 25 pathways (**C**) and upstream regulators (**D**), which show enhancement (positive *z* scores, red) or suppression (negative *z* scores, blue), in HPgV^–^ PD whole blood as compared with CT (left column) or in HPgV^+^ PD whole blood as compared with CT (right column). Ranked in order of absolute value, from lowest cumulative rank score to highest. Italicized pathways/regulators indicate an enhancement of the PD phenotype as determined by a more extreme *z* score in the HPgV^+^ as compared with HPgV^–^ analysis. Pathways/regulators that were also altered in brain tissue are marked with an asterisk.

*FUBP3*, a nucleic acid binding protein known to interact with the flavivirus JEV and limit neuronal activation of NLRP3 inflammasome (30, 31), was decreased in PD HPgV^–^ patients compared with other groups, but it was similar in PD HPgV^+^ and CT (Figure 4A, adjusted P (*P*_adj_) = 0.005). *TSC22D3*, a transcriptional regulator of antiinflammatory genes that is also known to inhibit NF-κB activity, was found to be increased in PD HPgV^–^ patients relative to other groups (Figure 4B, *P*_adj_ =0.012) (32).

The top 25 affected pathways and upstream regulators for HPgV^–^ PD and HPgV^+^ patients with PD from whole blood, compared with matched CT, are shown in Figure 4, C and D.

### Longitudinal analysis of HPgV viremia in patients with PD and CT.

**Figure 5:**
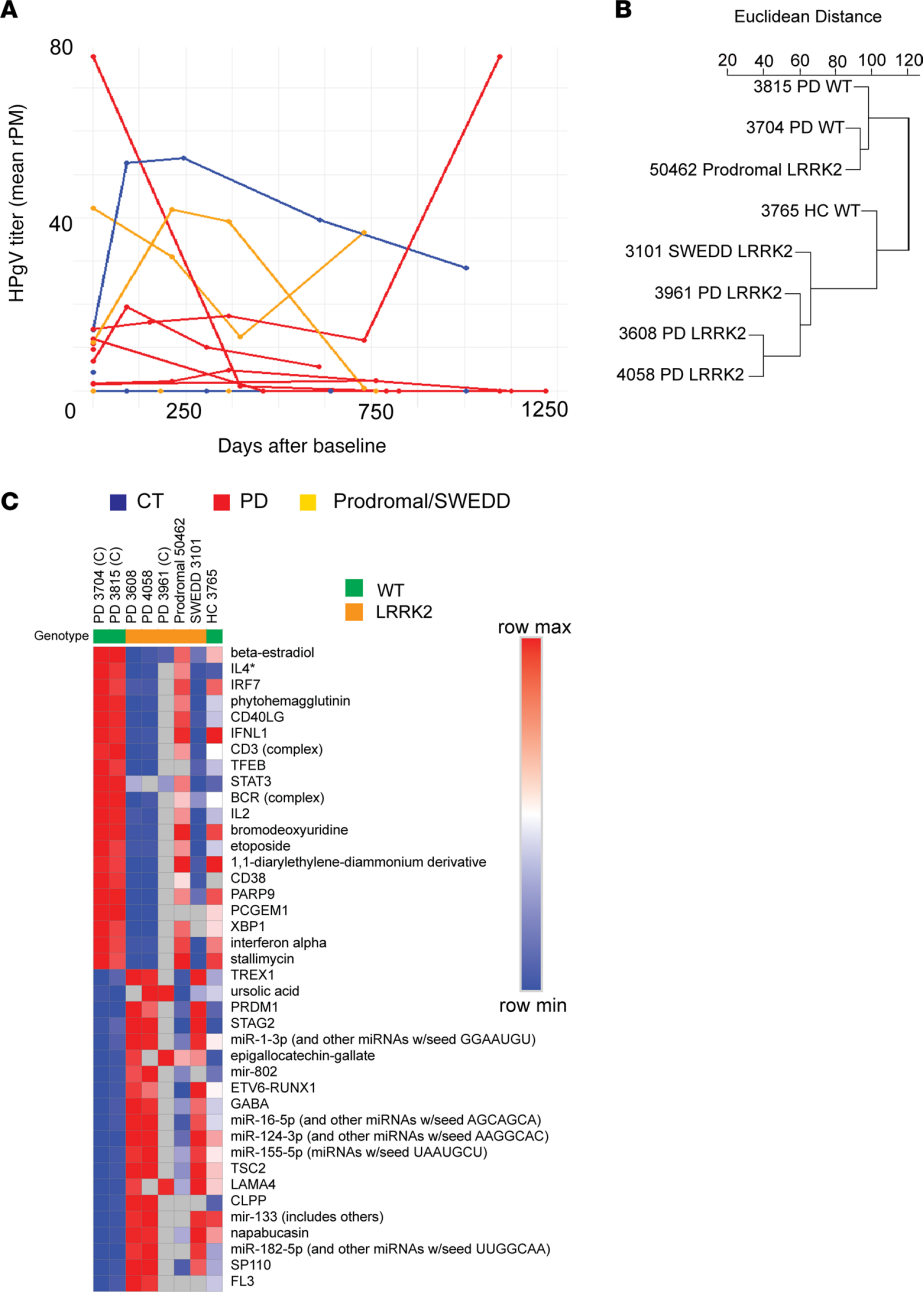
HPgV persistence and transcriptomics associations in whole blood of patients with PD and controls over time. (**A**) Line plot of mean HPgV titers (rPM, from 4 sequencing lanes) from all available longitudinal samples colored by diagnostic grouping (CT, blue; PD, red; SWEDD/prodromal, orange). (**B**) Clustered dendrogram of Euclidean distance between expression profiles (lower scores more similar) relative to HPgV titer shows genotypic separation between PD groups. (**C**) Heatmap of top upstream regulators that are most divergent between PD-WT and PD-LRRK2 patients, showing enhancement (positive *z* scores, red) or suppression (negative *z* scores, blue). Gray boxes indicate no significant pathway/regulator change between conditions. Ranked in order of S2N ratio, top 20 positive and negative markers are shown. Patients followed by “(C)” had a cleared HPgV infection before the final time point available. IL-4 previously identified as an upstream regulator in brain and blood is marked with an asterisk.

Whole blood transcriptomic analysis was performed periodically for PPMI patients and was used to determine the kinetics of HPgV in whole blood for PD and other groups over time. All available time points from HPgV^+^ patients were obtained and analyzed through the ViroFind bioinformatics pipeline. Patients from all 4 groups were available with final time points between 609 and 1,218 days after the baseline sample. Viral persistence was observed in all groups through > 2 years following the baseline measurement, while 5 of 11 (45%) patients who had longitudinal data available achieved viral clearance during the study period (PD, 3 of 6; prodromal, 0 of 1; SWEDD, 1 of 2; CT, 1 of 2; Figure 5A). This analysis suggests that disease status does not correspond to changes in viral kinetics over the period of 2 years in the periphery.

### Transcriptomic associations with HPgV viremia in patients with PD and CT.

We analyzed transcriptomic associations with HPgV viremia to identify genes correlated with viral titer in HPgV^+^ patients who had 3 or more time points available. Viral titer was determined using HPgV rPM from the same sample, as identified through the ViroFind pipeline. Pearson correlations were calculated for each gene to identify transcriptional changes associated with HPgV in 5 patients with PD and 3 non-PD CT (1 CT, 1 SWEDD, 1 prodromal patient).

When hierarchically clustered, patients with PD carrying a *LRRK2* mutation (PD-*LRRK2*, *n* = 3) and those without the mutation (WT; PD-WT, *n* = 2) separated distinctly by Euclidean distance (Figure 5B). This finding suggests that *LRRK2* genotype influences transcriptional responses to HPgV infection, prompting us to perform a comparative analysis of PD-WT and PD-*LRRK2* patients to identify affected upstream regulators (Figure 5C).

IL-4, a key upstream regulator, was consistently decreased in HPgV^+^ patients with PD compared with CT in both brain and whole blood. However, its correlation with HPgV titer was genotype specific: in PD-*LRRK2* HPgV^+^ patients, IL-4 signaling decreased as HPgV titer increased, whereas in PD-WT HPgV^+^ patients, IL-4 signaling increased with rising HPgV titer. These opposing responses indicate that the presence of the *LRRK2* mutation alters how immune signaling pathways interact with viral load, highlighting a genotype-specific role for *LRRK2* in modulating immune responses to HPgV infection.

### Gene network analysis reveals YWHAB as a central node of genes differentiating PD-WT and PD-LRRK2 responses to HPgV infection.

**Figure 6:**
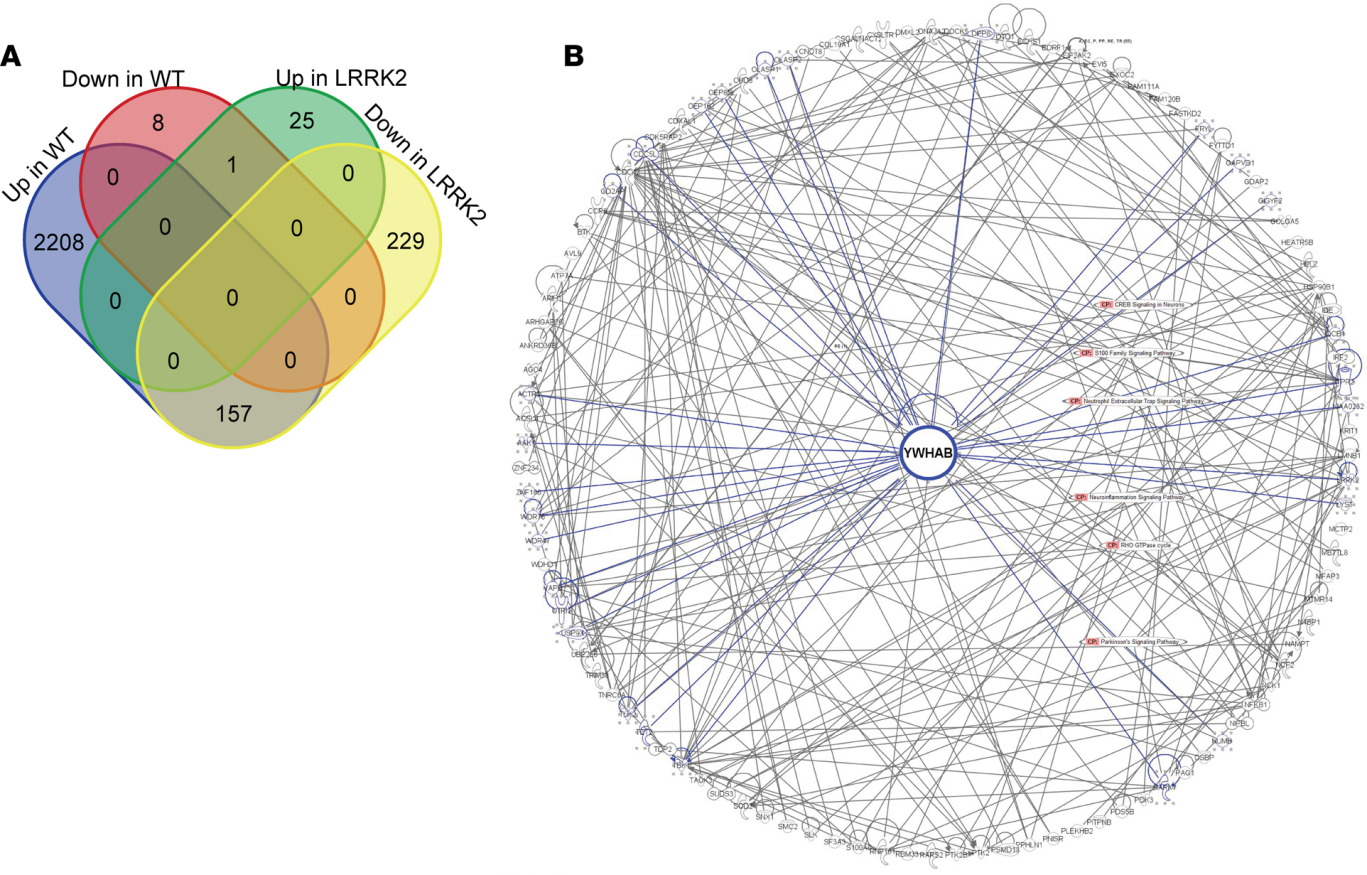
Differential gene expression and interaction network centered on *YWHAB* in PD-WT and PD-*LRRK2* responses to HPgV infection. (**A**) Venn diagram showing the pattern of genes differentiating PD-WT and PD-*LRRK2* responses to HPgV infection. (**B**) Radially aligned gene interaction network centered on *YWHAB*. Blue edges highlight direct interactions with *YWHAB*. Select canonical pathways are overlaid to demonstrate functional relationships among the connected genes (e.g., regulation, coexpression, or protein binding).

We then sought to identify genes that had significant associations with HPgV titer based on WT or *LRRK2* genotype. All genes with an unadjusted *P* < 0.05 by Pearson correlation, but opposing direction in PD-WT and PD-*LRRK2*, were compared (Figure 6A). The small nucleolar RNA (snoRNA), *SNORA45A*, was significantly negatively associated with HPgV titer in PD-WT patients but positively associated with HPgV titer in PD-*LRRK2* patients. Interestingly, all significant positive associations with HPgV titer in PD-*LRRK2* patients were from the *SNORA* class: *SNORA8, SNORA24, SNORA2A, SNORA10, SNORA71D, SNORA31, SNORA71B, SNORA26, SNORA43, SNORA34, SNORA16A, SNORA54, SNORA70, SNORA68, SNORD15A, SNORA21, SNORA80B, SNORA80A, SNORA7A, SNORA17, SNORA48, SNORA9, SNORA62, SNORA76A,* and *SNORA7B*. We identified a group of 157 genes that were positively associated with HPgV titer in PD-WT but significantly negatively associated with HPgV titer in PD-*LRRK2* (Figure 6B). These genes clustered around the 14-3-3 gene Tyrosine 3-monooxygenase/tryptophan 5-monooxygenase activation protein β (*YWHAB*) when assembled into an Ingenuity Pathways Analysis (IPA) gene interaction network, suggesting a pivotal role for this gene in the differential response to HPgV in PD-WT and PD-*LRRK2* patients.

### YWHAB expression predicts differences in LRRK-PD and PD-WT response to HPgV infection.

**Figure 7:**
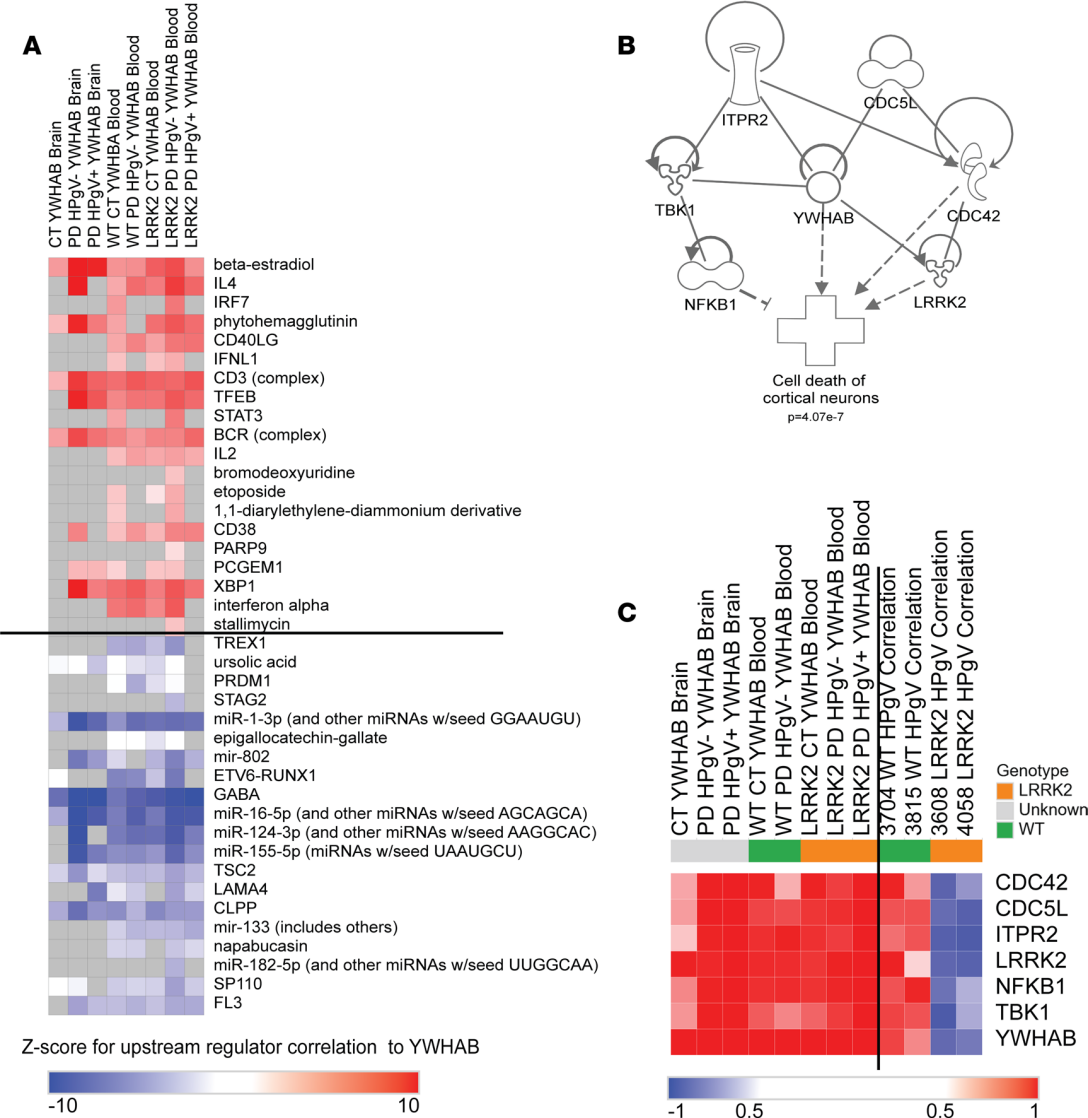
*YWHAB* expression correlates to HPgV response in PD-WT and PD-*LRRK2* in both brain and blood. (**A**) Heatmap of summary *z* score for suppression (blue) or enrichment (red) for upstream regulators by Pearson’s correlation to *YWHAB* in brain and blood transcriptomes. Regulators that were previously identified as differentiating between the response to HPgV in PD-WT and PD-*LRRK2* are shown to correlate with *YWHAB* expression in PD and Non-PD groups. Upstream regulators that were identified as enhanced by HPgV in PD-WT and repressed in PD-*LRRK2* (above black line), as compared with those that were repressed by HPgV in PD-WT but enhanced in PD-*LRRK2* (below black line). (**B**) Gene interaction network of the 7 genes that maintain the interaction network, which differentiates between the PD-WT and PD-*LRRK2* response to HPgV and their relationship to “cell death of cortical neurons” (*P* = 4.07 × 10^–7^). Nodes represent specific genes, and edges represent different types of functional interactions: Solid arrows indicate direct interaction, broken lines indicate an indirect interaction, blunted end indicates inhibition, and unended lines indicate nontargeting interactions. (**C**) Heatmap of group-based gene expression relative to *YWHAB* (left of black line) shows consistency of relationship between genes in all patient groups and tissue types, but they show disruption in relationship to HPgV in PD-WT and PD-*LRRK2* from longitudinal analysis (right of black line)

To validate the identification of *YWHAB* as the central hub distinguishing responses between PD-*LRRK2* and PD-WT, we analyzed genes that covary with *YWHAB* across samples from the brain and whole blood between donors rather than within the same donor as was performed in the longitudinal analysis. Figure 7 demonstrates that *YWHAB*’s interactions with the previously identified significant upstream regulators are preserved across different PD and CT subtypes and tissues, reinforcing its central role. In both blood and brain compartments, *YWHAB* consistently covaries with upstream regulators identified in Figure 5 to differentiate PD-WT (*YWHAB*^hi^) and PD-*LRRK2* (*YWHAB*^lo^) responses, showing positive *z* scores for the top 20 upstream regulators (Figure 7A, above black line), which were previously found to be increased by HPgV in PD-WT but decreased in PD-*LRRK2*, and vice versa (Figure 7A, below black line). Additionally, β-estradiol, CD3 (complex), BCR (complex), miR-1-3p, GABA, miR-16-5p, TSC2, and CLPP exhibit significant associations with *YWHAB* across all groups in both brain and blood compartments, with decreased GABA responses being the most significant association with *YWHAB* out of not only these regulators but all regulators in the database. Next, we removed *YWHAB* from the gene network to establish which other participating genes were able to function as central hubs. Six additional genes were identified that functioned as central hubs before the network lost centrality. In order, these were: *CDC42*, *TBK1*, *CDC5L*, *LRRK2*, *NFKB1*, and *ITPR2* (Figure 7B). IPA for disease and functional associations with these genes yielded “Cell Death of Cortical Neurons” as the most consistent association (*P* = 4.07 × 10^–7^). A heatmap of these genes for group wise *YWHAB* correlations (Figure 7C, left of black line) and individual HPgV longitudinal correlations (Figure 7C, right of black line) shows that *YWHAB* as an anchor predicts the expression of the remaining 6 genes regardless of genotype, infection status, disease status, or tissue type. The stability of these associations further validates *YWHAB* expression as a central node in this network which effectively differentiates the HPgV responses between PD-*LRRK2* and PD-WT.

## Discussion

Our findings provide insights into the interaction between HPgV infection and PD, both in the CNS and peripheral blood. Collectively, we have shown that HPgV is present in the CNS and whole blood of patients with PD, that patients with HPgV nucleic acids in the brain have more advanced NFT pathology and complexin-2 protein levels, and that those with HPgV nucleic acids in the whole blood have higher IGF-1 and lower pS65-ubiquitin concentrations in the plasma. We found that HPgV infection in patients with PD induces marked transcriptomic alterations, particularly affecting IL-4 signaling. In the absence of HPgV infection, IL-4 functioned as an enhanced upstream regulator in both the brain and whole blood of patients with PD compared with CT. However, in HPgV-infected patients with PD, regulation by IL-4 became significantly negative. We have also shown that, among patients with PD with HPgV infection, IL-4 signaling significantly changed with HPgV titer in a genotype-dependent manner. The response to HPgV infection involved a network of 157 genes that were upregulated in PD-WT relative to HPgV titer but was downregulated in PD-*LRRK2* patients relative to HPgV titer. Central to this network was the *YWHAB* gene, which exhibited opposing patterns of regulation between the 2 genotypes. We additionally showed that *YWHAB* expression independently predicted the upstream regulator alterations seen in the different response to HPgV based on genotype in all patient groups and tissue types, and 6 additional key regulators that functioned as central hubs to the differential responses between genotypes were identified.

### HPgV prevalence and its association with PD pathology.

HPgV (formerly known as GB virus C or Hepatitis G Virus) is a panlymphotropic, enveloped, single-stranded, positive sense RNA flavivirus that is closely related to the Hepatitis C virus based on genome homology. While poorly studied, it is thought that HPgV infection is typically subclinical. Persistent viremia over years to decades in healthy blood donors has been observed in 1%–5% of the population (33–35). Among other mechanisms, HPgV maintains persistence by dampening immune activation through inhibiting the activity of cytokines by direct inhibition of signaling cascades down stream of Tyk2 (36). HPgV sequence has often been detected in brains and CSF from those infected with HIV and has been found in biopsies from encephalitis with T cell infiltrations. However, infection of brain parenchymal cells has only been reported infrequently, including in individuals presenting with leukoencephalitis (28, 37, 38).

In our study, HPgV was identified in 50% of PD brain tissue samples, with no detection in CT brain tissue, suggesting an enrichment of HPgV infection in the brains of patients with PD, and HPgV protein expression was confirmed in 2 patients with PD tested. Although HPgV^+^ and HPgV^–^ patients with PD had similar demographic and clinical features, HPgV^+^ patients exhibited more advanced limbic Braak staging of NFT, indicating that HPgV might contribute to, or be correlated with, more severe neuropathology rather than being causal. Alternatively, more severe pathology in PD could facilitate a greater frequency of HPgV neuroinvasion, warranting further exploration of this relationship. Furthermore, higher levels of complexin-2 in HPgV^+^ patients suggest an alteration in synaptic function, particularly in glutamate release. Altered immune responses, including peripheral immune dysregulation and chronic neuroinflammation, are increasingly recognized as central to PD pathogenesis (39), and HPgV infection may contribute to these processes. However, further studies are needed to determine the exact role of HPgV in immune dysregulation and its potential effect on disease progression. These findings imply that HPgV may influence or exacerbate synaptic dysfunction and tau pathology in PD. Notably, the absence of HPgV in CT brains, combined with its presence in PD brains despite its well-documented persistence in blood, raises important questions about the factors influencing its detection in the CNS. Given that HPgV does not establish latency, its presence in brain tissue suggests active replication. HPgV has been identified to replicate in glial cells, though whether this occurs in the context of PD remains unknown. Host-related factors such as blood-brain barrier dysfunction or neuroinflammatory changes could influence its distribution. In addition, immune changes associated with PD pathogenesis may enable HPgV neuroinvasion and persistence. Further studies with larger datasets and mechanistic approaches will be necessary to determine the relevance of HPgV in the CNS and its potential relationship to disease pathology.

### Immune signaling and transcriptomic effects in the CNS and blood.

Our findings demonstrate that HPgV infection is associated with alterations in immune signaling and transcriptomic profiles in both the CNS and peripheral blood of patients with PD. Although HPgV prevalence in blood did not significantly differ between PD and CT groups, transcriptomic analyses revealed distinct immune responses associated with HPgV infection in peripheral blood. These findings suggest that, while viremia alone does not distinguish PD from CT, systemic immune alterations associated with HPgV may still be relevant to disease mechanisms. Notably, the separation between PD-WT and PD-*LRRK2* patients became apparent during Pearson correlation analyses with HPgV titer, where clustering patterns indicated distinct immune regulatory differences between genotypes. Given that *LRRK2* mutations are known to influence immune signaling, autophagy, and viral processing, these genotype-specific transcriptomic responses suggest that host genetic background, and virus host interactions, may shape the immune response to HPgV in a manner relevant to neuroinflammation and PD pathogenesis. While the implications of these differences require further study, they highlight the potential for genetic factors to influence pathogen-host interactions in PD. HPgV-associated pathways included IL-4 and IFN-γ signaling, which exhibited genotype-dependent responses, indicating that immune modulation by HPgV varies depending on host genetic background and tissue compartment. We also found that HPgV infection is associated with genes linked to PD and Parkinsonism, such as increased MMP9 signaling in the brain and enhanced EIF2AK2 signaling in the peripheral blood, suggesting potential mechanisms by which viral infection may exacerbate neuroinvasion and neuroinflammation through extracellular matrix remodeling and cellular stress responses (40–42).

HPgV also appears to differentially affect the expression of snoRNAs based on genotype. For example, increased *SNORA* expression was observed in PD-*LRRK2* patients but decreased in PD-WT patients, suggesting a genotype-specific role for snoRNAs in modulating immune responses and neuroinflammatory processes. These findings align with the known role of snoRNAs in systemic inflammation and mitochondrial dysfunction, which are central features of neurodegenerative diseases such as PD (42).

Significant compartment-specific effects were observed in immune-related pathways. Neutrophil degranulation was suppressed in the CNS of patients with PD, with further suppression in HPgV^+^ individuals, while it was enhanced in the peripheral blood of patients with PD and further amplified in those with HPgV infection. Other pathways, such as IL-4/IL-13 signaling and TREM1 signaling, were affected only in the brain, reflecting localized immune dysregulation. In contrast, peripheral effects included activation of interferon α/β signaling, consistent with prior reports of HPgV-induced ISG expression associated with IFNL1, IFNA2, and IFNG in PBMCs (43). However, MAVS cleavage by HPgV NS3/4AB has been shown to inhibit type I IFN responses by preventing IFN-β transcription (44), suggesting that HPgV’s modulation of interferon signaling may vary depending on context.

HPgV-mediated alterations also involved pathways critical to neuronal function, signaling, and inflammation. Pathways such as CREB signaling in neurons, cAMP-mediated signaling, G-protein coupled receptor (GPCR) signaling, and S100 family signaling, which are all linked to PD pathology, were enhanced in patients with PD but significantly suppressed in HPgV^+^ patients with PD (43–46). This suppression suggests that HPgV infection may disrupt neuronal homeostasis and communication, potentially exacerbating PD pathogenesis.

In a study of an HIV^+^ cohort, HPgV^+^ individuals were found to have decreased IL-4 levels while IL-2 levels were unaffected (45). This contrasts with another study, which reported increased IL-2 and IL-17A levels in peripheral blood but no changes in IL-4, IL-10, or TNF-α levels in individuals who are viremic (47). Our findings of altered IL-4 signaling in patients with PD suggest that HPgV’s effects on cytokine regulation may depend on the host’s disease context and genetic background, highlighting the complex interplay between HPgV and immune pathways in different populations.

### The role of IL-4 signaling, YWHAB, and immune dysregulation.

Critical findings of this study include the genotype-dependent effect of HPgV infection on IL-4 signaling and the *YWHAB* (14-3-3 β) gene network—specifically, increasing HPgV titer results in opposing regulatory effects in the detection of IL-4 as an upstream regulator and differential expression of *YWHAB* and its interacting partners between PD-WT and PD-*LRRK2* genotypes. In PD-WT, IL-4 is found to be enhanced as an upstream regulator, and *YWHAB*, *CDC42*, *TBK1*, *CDC5L*, *LRRK2*, *NFKB1*, and *ITPR2* are upregulated with increasing HPgV titer, whereas in PD-*LRRK2*, IL-4 signaling and these genes are downregulated. These opposing patterns of regulation for the hub genes have direct implications for the “cell death of cortical neurons” pathway, which is found to be suppressed when these genes are upregulated and enhanced when they are downregulated in this interaction network. This suggests that HPgV infection disrupts neuronal homeostasis through genotype-specific modulation of signaling networks. TBK1, which is upstream of NF-κB signaling, is known to interact with YWHAB and is involved in inflammatory signaling (48). NF-κB1 is a key transcription factor that regulates the expression of genes involved in inflammation and cell survival. The dysregulation of NF-κB signaling has been linked to neuroinflammation in PD, and YWHAB’s role in modulating this pathway could be crucial for neuronal protection (49). Similarly, TBK1 is involved in autophagy and inflammatory responses, and its interaction with YWHAB may influence the cellular response to stress and inflammation (50). YWHAB and ITPR2 interactions play a role in calcium signaling, which is vital for neuronal functions, including neurotransmitter release and synaptic plasticity. Dysregulation of calcium homeostasis is a hallmark of neurodegenerative diseases, and YWHAB’s involvement in calcium signaling pathways may be critical for maintaining neuronal health (51, 52). YWHAB additionally directly interacts with CDC42 and LRRK2, both of which are crucial for cytoskeletal dynamics and neuronal morphology (53). YWHAB’s role in modulating LRRK2 activity through binding of the WD40 domain activity could have implications for dopaminergic neuron survival and function (54). The differential effect of HPgV infection on these pathways between WT and *LRRK2* genotypes highlights a potentially novel mechanism by which viral infections interact with genetic risk factors to modulate neurodegenerative processes in PD.

Our findings indicate that HPgV infection modulates signaling pathways in a genotype-specific manner, aligning with genes known to inherently differ in PD-*LRRK2* and PD-WT. STAT3 and TFEB signaling increased with HPgV titer in PD-WT but decreased in PD-*LRRK2*, consistent with prior reports that *LRRK2* mutations, particularly G2019S, influence STAT3-mediated immune regulation and TFEB-driven lysosomal function (55, 56). In contrast, TSC2 signaling decreased with HPgV titer in PD-WT but increased in PD-*LRRK2*, which is notable given TSC2’s role in mTOR regulation, a pathway disrupted in *LRRK2*-associated PD. Additionally, *YWHAB* (14-3-3 β), a known regulator of LRRK2, exhibited genotype-dependent modulation with HPgV infection. The interaction between LRRK2 and 14-3-3 proteins plays a crucial role in maintaining LRRK2 in an inactive conformation, but PD-associated *LRRK2* mutations destabilize this interaction, leading to increased kinase activity (57). Our findings suggest that HPgV infection may further influence this regulatory mechanism, potentially exacerbating immune dysregulation and neurodegenerative processes in PD-*LRRK2*. While these findings highlight the interaction between viral infection and genotype-specific signaling pathways, the relevance of these changes remains to be determined.

### Limitations.

This study has a number of limitations. First, the number of HPgV^+^ patients in all groups was relatively small, which affects the generalizability of our findings. To assess the robustness of our findings, we conducted post hoc power analyses for 2 key aspects of our study: the qualitative detection of HPgV positivity in brain tissue and the analysis of IL-4 signaling as an upstream regulator.

For HPgV positivity, we evaluated the post hoc power to detect differences between PD and CT brain samples across all CNS groups. In our dataset, 50% of PD brain samples (5 of 10) were HPgV^+^ compared with 0% of CT brain samples (0 of 14). This analysis yielded a power of approximately 85.9%, reflecting strong sensitivity to detect this difference, even with the small sample sizes. For IL-4 signaling, we analyzed the power to detect differences between HPgV^+^ PD, HPgV^–^ PD, and HPgV^–^ CT groups. In brain data, the power to detect a difference between HPgV^+^ PD (*n* = 3) and HPgV^–^ CT (*n* = 4) with an observed effect size of 3.91 was nearly 100%. In contrast, the comparison between HPgV^–^ PD (*n* = 4) and HPgV^–^ CT (*n* = 4) with a smaller effect size of 0.718 had limited power (42%). In blood data, the power to detect a difference between HPgV^+^ PD (*n* = 7) and HPgV^–^ CT (*n* = 21) with an observed effect size of 1.88 was approximately 99%, while the comparison between HPgV^–^ PD (*n* = 21) and HPgV^–^ CT (*n* = 21) with an effect size of 4.281 had nearly 100% power.

These analyses highlight that, while the study is well powered to detect large differences in HPgV positivity and IL-4 signaling, smaller effects, particularly in brain samples, may require larger cohorts for adequate sensitivity. Future studies with expanded sample sizes will be essential to confirm and extend these findings. HPgV is challenging to screen for, as there are no commercially available detection tests nor quantitated standards, and it is typically a subclinical infection. Despite this, we believe our qualitative results are relevant because they provide insights into the role of HPgV in PD, particularly in the context of genotype-specific and compartmentalized immune responses. Additionally, while our results suggest consistent changes across both compartments (CNS and blood), the effects of HPgV in the periphery and CNS may differ, warranting further study.

Another limitation is the lack of genotyping data for the brain tissue samples, which prevented us from stratifying HPgV responses based on WT or *LRRK2* genetic background in the CNS. Furthermore, we could not determine whether HPgV infection occurred before or after the onset of PD symptoms, as this study was retrospective. Retrospective designs inherently limit the availability of detailed clinical data, such as duration of infection or viral load over time. Future prospective studies are needed to address these questions.

We also acknowledge that the differences in mean ages between the brain tissue and blood cohorts reflect the inherent design of the respective studies; brain tissue is collected postmortem, whereas the PPMI blood cohort is designed as a longitudinal study enrolling participants earlier in life. These age differences may introduce variability in immune function and transcriptomic profiles between cohorts. While our study was not designed to directly assess age-related effects, we note this as a potential limitation and encourage future studies to investigate the interaction between age, viral infection, and PD pathology in more detail.

### Conclusion.

In summary, our results indicate that HPgV infection is associated with substantial transcriptomic and immune signaling changes in patients with PD, particularly affecting pathways related to IL-4 signaling as well as signaling related to neuroinflammation and neuronal function. Many changes appear to be genotype dependent, with *LRRK2* mutation carriers showing distinctly different responses to HPgV infection, possibly due to LRRK2 involvement in immune responses. The modulation of *YWHAB* and its gene network shows a genotype-dependent response to HPgV that suggests these changes may be associated with death of cortical neurons. Thus, HPgV and viral infections may influence PD etiology, progression, or pathogenesis. Future studies are needed to further elucidate the role of HPgV in PD and to explore whether targeting viral infections or associated immune pathways could provide therapeutic benefits.

## Methods

### Sex as a biological variable

Our study examined female and male patients with PD and CT, and findings are reported for both sexes. Data analyses were performed to determine whether study results were related to sex by performing statistical analyses determining whether there were sex differences in viral prevalence and titers (Table 4 and Supplemental Table 1). All RNA-Seq analyses used sex-matched reference cases.

### Clinical samples

Deidentified fresh frozen and contralateral FFPE postmortem brain tissue from the AMG, PPUT, and SFC of 10 PD and 14 age- and sex-matched CT was obtained from the RADC. Matched plasma and CSF were also provided.

### ViroFind sample processing

ViroFind processing was performed similar to previous publications (26). Protocol changes included using Qiagen Qiashredder followed by RNeasy for RNA extraction, Agilent SureSelect XT2 Target Enrichment System for Illumina sequencing hybridizing with biotinylated 125 nucleotide long RNA probes designed against the full genome length sequences of all viruses known to infect humans, and those that are considered to have zoonotic potential (ViroFind probes; ref. 26) for 24 hours at 65°C. Deep sequencing for virus enriched samples was performed using the Illumina NextSeq sequencer at Northwestern University NUSeq. Each sample had about 20 million paired-end (PE) 150 bp reads.

### ViroFind bioinformatics pipeline

Raw demultiplexed reads were processed with the previously published ViroFind pipeline (v2.0) (26). Reads with quality < 20 or length < 50 bp were removed using Skewer, followed by low-complexity and duplicate filtering with PRINSEQ++. High-quality reads were mapped to the human genome using BWA-MEM; unmapped reads were aligned to a curated database of viral genomes from NCBI. Reads mapping ambiguously to multiple viruses were excluded. Viral coverage metrics were calculated with BEDTOOLS, and alignment files (SAM/BAM) were generated for visualization. Viral regions were annotated using corresponding GFF files with BEDOPS and custom scripts. Per-sample summary files reported detected viruses and normalized breadth/depth of coverage (per million reads).

### qPCR assay

An in-house qPCR assay was developed to detect HPgV nucleic acid due to the lack of commercial assays. Two genomic regions (NS3 and 5′UTR) were targeted using degenerate primers and triplicate TaqMan probes to capture viral sequence diversity. Primer pairs were validated using a full-length molecular clone of HPgV (NIH HIV Reagent Program, ARP-9450), which clusters with genotype 2 and is cloned into pCR2.1 (35). The plasmid was spiked into human genomic DNA to confirm specificity and rule out nonspecific amplification, with and without degenerate primers.

Validation was repeated using RNA transcribed from linearized plasmid (SpeI digestion) with the T7 RiboMAX system (Promega). Transcribed RNA was spiked into extracted human DNA and amplified using TaqMan Fast Virus 1-Step Master Mix to confirm RNA-template specificity and the absence of off-target amplification. Final qPCR validation was performed on the QuantStudio 5 (Thermo Fisher Scientific) using TaqMan probes. HPgV RNA was diluted to 5 ng/μL (approximately 1 × 10^9^ copies/μL), and serial 10-fold dilutions established a linear dynamic range from 1 × 10^5^ to 1 × 10² copies per reaction.

Primer and probe sequences (listed 5′–3′) were as follows: NS3 forward primers GTGACCTGGTATGGAATGGAACCT and GTAACCTGGTATGGAATGGAACCT, reverse primer CGACGGCTGCGGTGTAAG, and probes 6FAM-AGCTAACCTATTGAGACTTTACGACGACT-QSY, VIC-AGCTAACCTACTGAGACTTTACGACGACT-QSY, and ABY-AGCTAACCTTCTGAGACTTTACGACGACT-QSY. 5′-UTR forward primer TGTTGGCCCTACCGGTGTTA, reverse primer CCGTACGTGGGCGTCGTT, and probes 6FAM-CTCGTCGTTAAACCGAGCCCGTCA-QSY, VIC-CTCGTCGTTAAACCGAGACCGACA-QSY, and ABY-CTCGTCGTTAAACCGAGACCGTCA-QSY.

qPCR was performed in triplicate using 10 μL of extracted RNA per reaction. Cycling conditions were 50°C for 10 minutes (reverse transcription), 95°C for 20 seconds (enzyme inactivation), followed by 40 cycles of 95°C for 15 seconds and 60°C for 1 minute. Each run included positive, no-template, and HPgV^–^ RNA CT. Samples were considered positive if both the NS3 and 5′UTR regions showed a Ct ≤ 38 for at least 1 probe across all 3 replicates.

### IHC staining

IHC staining was performed on the FFPE brain samples from the hemisphere contralateral to the one used for ViroFind experiments. HPgV antibody against NS5A (Novus NB100-93584; Hepatitis G Virus NS5A) diluted at 1:500 was used to stain 6 μm–thick sections of FFPE tissue on charged slides. Slides were baked in the oven at 60°C for 60 minutes before going through deparaffinization and rehydration. Antigen retrieval was achieved using a pH 6 retrieval buffer (Biocare Reveal Decloaker). Slides were cooled to room temperature and washed in TBS before neutralizing endogenous peroxidase (Biocare Peroxidase 1). Slides were then treated with a serum-free casein background block (Biocare Background Sniper) before preincubation in a 10% goat serum block for 60 minutes. Primary antibody was then added to the slides for overnight incubation at 4°C. After incubation, slides were washed well with TBS-T before incubating in HRP/AP polymer (Biocare MACH 2 Double Stain 2) for 30 minutes. Reaction products were visualized with red chromogen (Biocare Warp Red). Slides were then counterstained with hematoxylin, left to air dry, and mounted with xylene-based mounting media. Slides were reviewed and interpreted by 2 board-certified neuropathologists.

### PPMI samples

PPMI whole transcriptomes and genomes were collected, sampled, sequenced, and genotyped as previously described (58–60). Transcriptomes, genomes, demographic information, signs and symptoms, assay results, and cognitive information were downloaded from LONI Image and Data Archive (LIDA, downloaded April-August 2024). An in-house R script was written to select samples for inclusion based on age, sex, and genotype matching.

### RNA-Seq

Raw transcriptomes from 4 sequencing lanes were mapped using the nf-core RNA-Seq module (61). Gene-level counts were extracted from the mapped data and used as input for differentially expressed gene (DEG) analysis. For small-sample RADC brain tissue dataset, DEG was performed using the Kruskal-Wallis nonparametric test to account for small group sizes and nonnormal data distribution. For larger datasets, from the PPMI whole blood sample groups, variance-stabilized transformation normalization (V.T-normalized) was applied using DESeq2 (v1.30.1) in R (v4.0.3) with blind = FALSE, accounting for experimental design. Metadata files were generated with the factor group combined, which categorized samples into 3 groups: PD HPgV^+^, PD HPgV^–^, and CT. The transformed data were used to detect differences in expression patterns between groups.

To identify DEGs, 1-way ANOVA was performed on V.T-normalized transcripts to compare expression across the 3 groups, and *P* values and effect sizes (log_2_ fold-changes) were extracted from summaries for IPA. BH correction was applied to CT the FDR < 0.05.

#### Phylogenetic analysis and tree.

Nucleotide sequences were aligned using seqmagick to convert the input FASTA file into relaxed PHYLIP format to preserve full sequence names. Maximum likelihood phylogenetic analysis was performed using RAxML-NG under the GTR+G model, which incorporates the General Time Reversible substitution model with a Gamma distribution to account for rate heterogeneity across sites. A single ML tree search was conducted using raxml-ng --search1 --msa alignment.phy --model GTR+G --prefix my_tree. To assess node support, 100 bootstrap replicates were generated using raxml-ng --bootstrap --msa alignment.phy --model GTR+G --prefix bootstrap --bs-trees 100. A majority-rule consensus tree was constructed from the bootstrap replicates using raxml-ng --consense --tree bootstrap.raxml.bootstraps --prefix consensus_tree. The final tree was visualized using FigTree and the Interactive Tree of Life (iTOL). 

#### Linear correlation for longitudinal data.

Gene expression data from RNA sequencing was processed alongside HPgV rPM (HPgV titer) identified through the ViroFind pipeline. Pearson correlation analysis was performed for each individual. Differential expression analysis was conducted using the edgeR package. Gene expression data were normalized using calculated normalization factors, and a design matrix was constructed with HPgV titer as a continuous covariate. Dispersion was estimated, and a generalized linear model (GLM) was fitted. Differentially expressed genes were identified through a likelihood ratio test (LRT) and ranked.

#### Linear correlation for group data.

GSEA was performed on mapped RNA-Seq data from each group separately, using a gene (*YWHAB*) as a phenotype for Pearson correlation using GSEA v4.2.3 (build 10) to obtain ranked gene files. Ranked genes were used for IPA as fold change.

### Heatmaps

Pathway, upstream regulator, and gene expression data were analyzed and visualized using Morpheus (https://software.broadinstitute.org/morpheus). A heatmap was generated to compare expression levels/*z* scores between groups. The signal-to-noise (S2N) algorithm was employed to rank regulators based on their ability to distinguish between 2 conditions. The S2N score was calculated as the difference between group means divided by the sum of SDs across groups. To assess the significance of the observed differences, 20 permutations of the sample labels were performed, generating a null distribution of S2N scores for each gene. Positive S2N values indicated higher expression in one group, and negative values indicated higher expression in the other.

### Statistics

ViroFind discoveries, demographic, clinical, and pathology differences were evaluated using unadjusted *P* values due to the exploratory nature of the analysis (62, 63). Transcriptomic data were adjusted for multiple comparisons using the Benjamini-Hochberg (BH) method to identify differentially expressed genes. Summary values were tested for normality using the Kolmogorov-Smirnov test. Parametric data were reported as mean ± SD and compared using 2-tailed *t* tests or 1-way ANOVA. Nonparametric data were reported as median with quartile 1 (Q1) and Q3 and compared using Kruskal-Wallis or Wilcoxon rank-sum tests. Categorical variables were analyzed using Fisher’s exact test. Specific tests are indicated in figure and table legends.

RNA-Seq data from brain samples were analyzed using Kruskal-Wallis. PPMI whole blood transcriptomes were normalized using variance-stabilizing transformation (DESeq2 v1.30.1), followed by DESeq2 differential expression analysis with BH correction. Pearson correlation was used to assess gene expression versus HPgV titer in longitudinal samples. Group-level RNA-Seq analysis used YWHAB as the phenotype in GSEA (v4.2.3) (64) with ranked gene files analyzed using IPA (Qiagen).

GLMs were fitted using edgeR to assess gene expression in relation to HPgV titer. The Figure 4C heatmap used S2N ranking with 20 permutations to generate null distributions. Significance was defined as *P* or *P*_adj_ < 0.05.

Post hoc power analysis was performed for each comparison based on observed effect sizes, sample sizes, and α = 0.05

### Study approval

This study received prior approval by Northwestern University IRB (no. STU00211310).

### Data availability

Raw sequencing data of brain tissue have been deposited in the Sequence Read Archive under project no. PRJNA1265217. PPMI data can be accessed by permission from the Parkinson’s Progression Marker Initiative. Raw data values from statistical analyses are available in Supporting Data Values.

## Author contributions

Conceptualization was contributed by BAH, and IJK. Data curation was contributed by BAH, XD, and ZSO. Formal analysis was contributed by BAH and KC. Investigation was contributed by BAH, XD, AS, PJ, and RJC. Methodology was contributed by BAH, XD, AS, PJ, BB, and SJL. Writing was contributed by all authors.

## Supplementary Material

Supplemental data

Supporting data values

## Figures and Tables

**Table 1:**
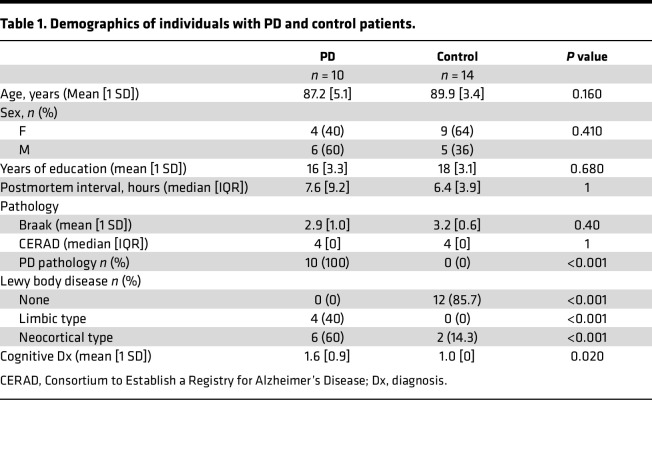
Demographics of individuals with PD and control patients.

**Table 2:**
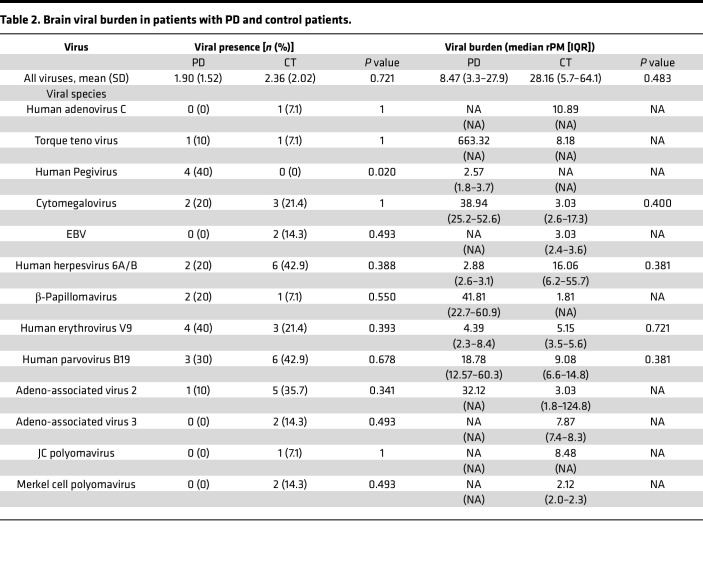
Brain viral burden in patients with PD and control patients.

**Table 3:**
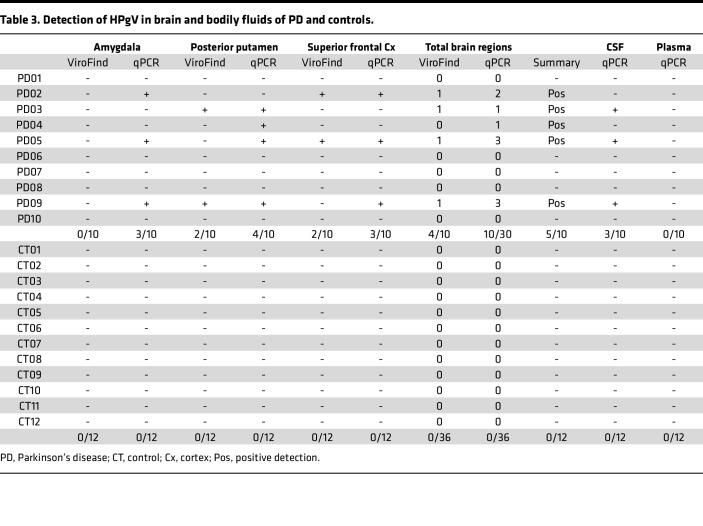
Detection of HPgV in brain and bodily fluids of PD and controls.

**Table 4:**
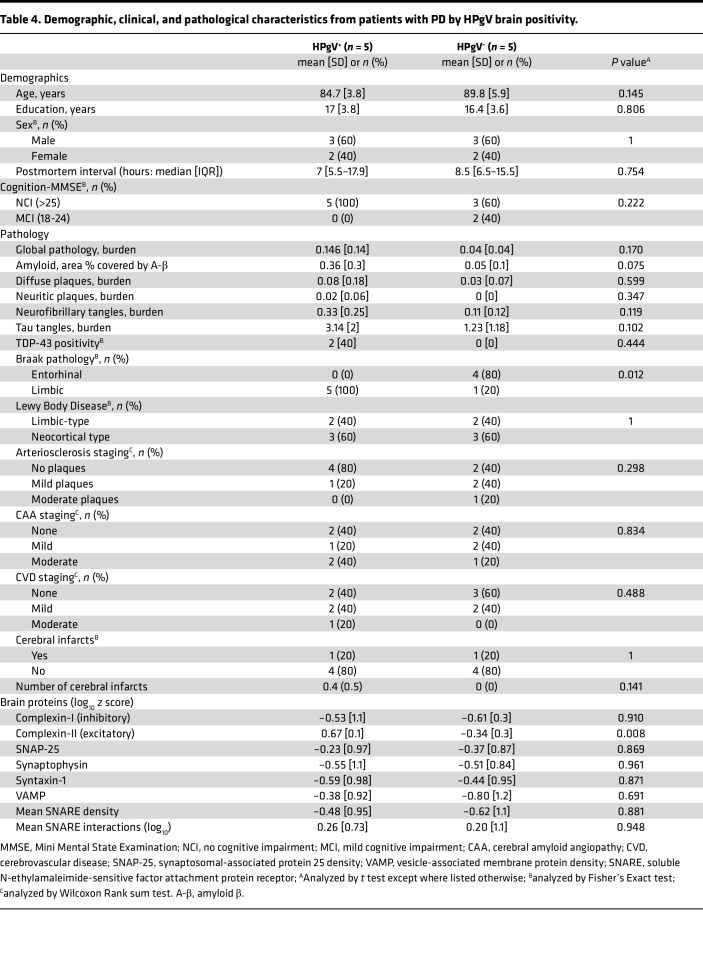
Demographic, clinical, and pathological characteristics from patients with PD by HPgV brain positivity.

**Table 5:**
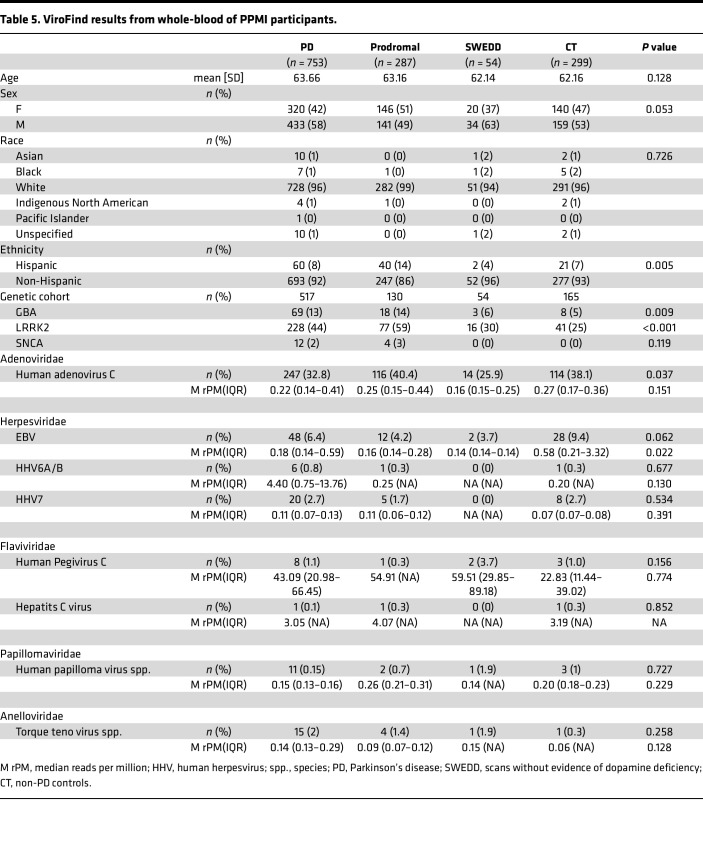
ViroFind results from whole-blood of PPMI participants.

**Table 6:**
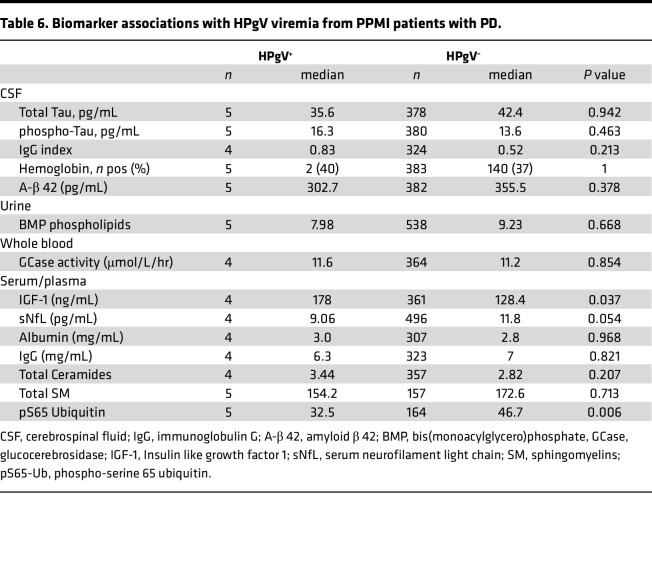
Biomarker associations with HPgV viremia from PPMI patients with PD.

## Version 1 07/08/2025

Electronic publication

## Version 2 07/10/2025

Steven J. Lubbe's name was corrected
