# Supplementary material for: Human pegivirus alters brain and blood immune and transcriptomic profiles of patients with Parkinson’s disease
Source: JCI Insight. 2025 Jul 8;10(13):e189988. doi: 10.1172/jci.insight.189988 (PMC12244338; doi:10.1172/jci.insight.189988)

Table S1: Demographics of PPMI patients by Viral positivity

	Total (n=1393)			PD (n=753)			Prodromal (n=287)			SWEDD (n=54)			CT (n=299)		
	HPgV -	HPgV +	p-value	HPgV -	HPgV +	p-value	HPgV -	HPgV +	p-value	HPgV -	HPgV +	p-value	HPgV -	HPgV +	p-value
Age, mean [1SD]	63.2	63.9	1	63.6	66.1	0.73	63.6	66.1	NA	62.3	58.0	0.518	62.2	62.1	1
Sex, n (%)															
F	620 (99)	6 (1)	1	318 (99)	2 (1)	0.478	145 (99)	1 (1)	1	19 (95)	1 (5)	0.608	138 (99)	2 (1)	0.601
M	759 (99)	8 (1)		427 (99)	6 (1)		141 (100)	0 (0)		33 (97)	1 (3)		158 (99)	1 (1)	
Genetic Cohort															
n=	856	10		512	5		129	1		52	2		163	2	
GBA	98 (11)	0 (0)	0.614	69 (14)	0 (0)	1	18 (14)	0 (0)	1	3 (6)	0 (0)	1	8 (5)	0 (0)	1
LRRK2	359 (42)	3 (30)	0.534	226 (44)	2 (40)	1	77 (60)	1 (100)	1	15 (29)	1 (50)	0.509	41 (25)	0 (0)	1
SNCA	16 (2)	0 (0)	1	12 (2)	0 (0)	1	4 (3)	0 (0)	1	0 (0)	0 (0)	NA	0 (0)	0 (0)	NA
	AdvC -	AdvC +		AdvC -	AdvC +		AdvC -	AdvC +		AdvC -	AdvC +	p-value	AdvC -	AdvC +	
Age, mean [1SD]	63.1	63.2	0.888	62.8	65.5	**<0.001**	64.7	60.9	**<0.001**	61.4	64.4	0.310	63.1	60.6	**0.012**
Sex, n (%)															
F	366 (62)	227 (38)	**0.027**	197 (62)	123 (38)	**0.006**	83 (57)	63 (43)	0.230	14 (70)	6 (30)	0.517	72 (51)	68 (49)	**<0.001**
M	540 (67)	260 (33)		309 (71)	124 (29)		90 (64)	51 (36)		27 (79)	7 (21)		114 (72)	45 (28)	
Genetic Cohort															
n=	672	194		398	119		89	41		41	13		144	21	
GBA	57 (9)	41 (21)	**<0.001**	39 (10)	30 (25)	**<0.001**	8 (9)	10 (24)	**0.027**	2 (5)	1 (8)	1	8 (6)	0 (0)	0.600
LRRK2	271 (40)	91 (47)	0.116	167 (42)	61 (51)	0.094	59 (66)	18 (44)	**0.021**	12 (29)	4 (31)	1	33 (23)	8 (38)	0.175
SNCA	4 (1)	12 (6)	**<0.001**	3 (1)	9 (8)	**<0.001**	1 (1)	3 (7)	0.092	0 (0)	0 (0)	NA	0 (0)	0 (0)	NA
	EBV -	EBV +		EBV -	EBV +		EBV -	EBV +		EBV -	EBV +	p-value	EBV -	EBV +	
Age, mean [1SD]	63.3	61.6	0.109	63.8	60.9	0.061	63.1	63.8	0.781	61.9	69.1	0.644	62.3	61.2	0.585
Sex, n (%)															
F	584 (93)	42 (7)	0.744	297 (93)	23 (7)	0.453	141 (97)	5 (3)	0.567	19 (95)	1 (5)	1	127 (91)	13 (9)	1
M	719 (94)	48 (6)		408 (94)	25 (6)		134 (95)	7 (5)		33 (97)	1 (3)		144 (91)	15 (9)	
Genetic Cohort															
n=	654	71		478	39		124	6		52	2		141	24	
GBA	88 (14)	10 (14)	0.856	63 (13)	6 (15)	0.629	17 (14)	1 (17)	1	2 (4)	1 (50)	0.107	6 (4)	2 (8)	0.329
LRRK2	341 (52)	20 (28)	**<0.001**	215 (45)	12 (31)	0.095	74 (60)	3 (50)	0.687	14 (27)	2 (100)	0.084	38 (27)	3 (13)	0.200
SNCA	16 (2)	0 (0)	0.389	12 (3)	0 (0)	0.613	4 (3)	0 (0)	1	0 (0)	0 (0)	NA	0 (0)	0 (0)	NA

Table S2: PD Clinical Characteristics by HPgV Positivity in Whole Blood

	HPgV -		HPgV +		Fisher's Exac
	Percent	Ratio / (n)	Percent	Ratio / (n)	p-value
Bradykinesis	80%	457/569	100%	5/5	0.589
Tremor	75%	431/575	100%	5/5	0.339
Rigidity	74%	420/566	100%	5/5	0.336
Response to Levodopa	47%	254/546	50%	2/4	1
Decreased arm swing	42%	219/522	50%	2/4	1
Micrographia	40%	210/530	50%	2/4	0.651
Dinimished olfaction	35%	186/525	50%	2/4	0.618
Motor Fluctuations	34%	185/545	50%	2/4	0.608
REM sleep disorder	24%	127/527	50%	2/4	0.250
Shuffling gait	30%	157/520	33%	1/3	1
Reflex assessment Abnormal	39%	228/583	20%	1/5	0.653
Stooped posture	33%	176/529	25%	1/4	1
Dyskinesis	26%	140/546	25%	1/4	1
Cranial Nerve II-XII assessment Abnormal	29%	130/447	20%	1/5	1
Coordination assessment Abnormal	8%	48/581	25%	1/4	0.296
Urinary dysfunction	33%	177/542	0%	0/4	0.310
Bowel dysfunction	28%	154/542	0%	0/4	0.581
Anxiety	17%	91/546	0%	0/4	1
Sexual dysfunction	16%	74/475	0%	0/4	1
Postural Hypotension	14%	74/528	0%	0/4	1
Depression	12%	66/546	0%	0/4	1
Apathy	11%	62/546	0%	0/4	1
Dystonia	11%	59/542	0%	0/4	1
Cognitive fluctuations	11%	58/540	0%	0/4	1
Dysphagia	11%	58/544	0%	0/4	1
Instability	10%	57/571	0%	0/5	1
Sensory Exam Abnormal	10%	56/581	0%	0/5	1
Seborrheic dermatitis	9%	48/517	0%	0/4	1
Dysarthia	6%	34/542	0%	0/4	1
No response to Levodopa	5%	22/468	0%	0/3	1
Prominent action tremor	4%	21/545	0%	0/4	1
Motor Exam Assessment Abnormal	3%	19/579	0%	0/5	1
Instability or gait issues in the first 3 years	3%	14/538	0%	0/4	1
Plantar-left Abnormal	2%	9/535	0%	0/4	1
Delusions/hallucinations	1%	7/544	0%	0/4	1
Myoclonous	1%	6/538	0%	0/4	1
Plantar-right Abnormal	1%	5/531	0%	0/4	1
Cortical sensory loss	0%	2/509	0%	0/4	1
Definite response to EtOH	0%	2/516	0%	0/4	1
Limb ideomotor apraxia	0%	2/535	0%	0/4	1
Disproportionate anterocollis	0%	2/543	0%	0/4	1
Inspiratory stridor	0%	2/544	0%	0/4	1
Neuroleptic super-sensitivity	0%	1/416	0%	0/3	1
Supranuclear gaze palsy	0%	1/538	0%	0/4	1
Wide gait/cerebellar features	0%	1/540	0%	0/4	1
Alien limb phenomenon	0%	0/541	0%	0/4	1
Pyramidal tract signs	0%	0/498	0%	0/4	1
Gait assessment Abnormal	0%	0/583	0%	0/5	1
Mental Status assessment Abnormal	0%	0/583	0%	0/5	1
Clinical global impression; mean	2.9	n=200	3.5	n=3	0.518

Supplemental Figure 1

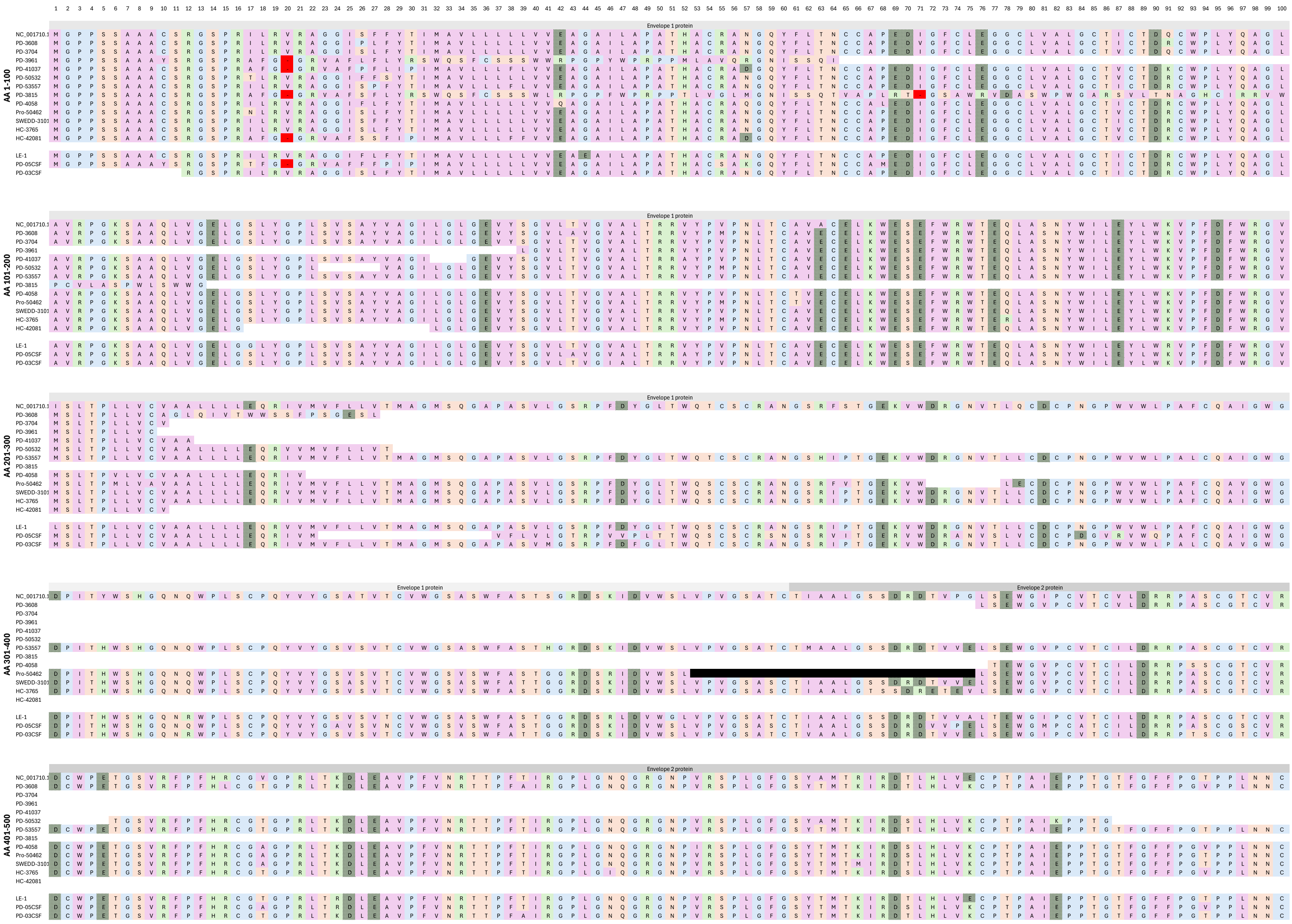

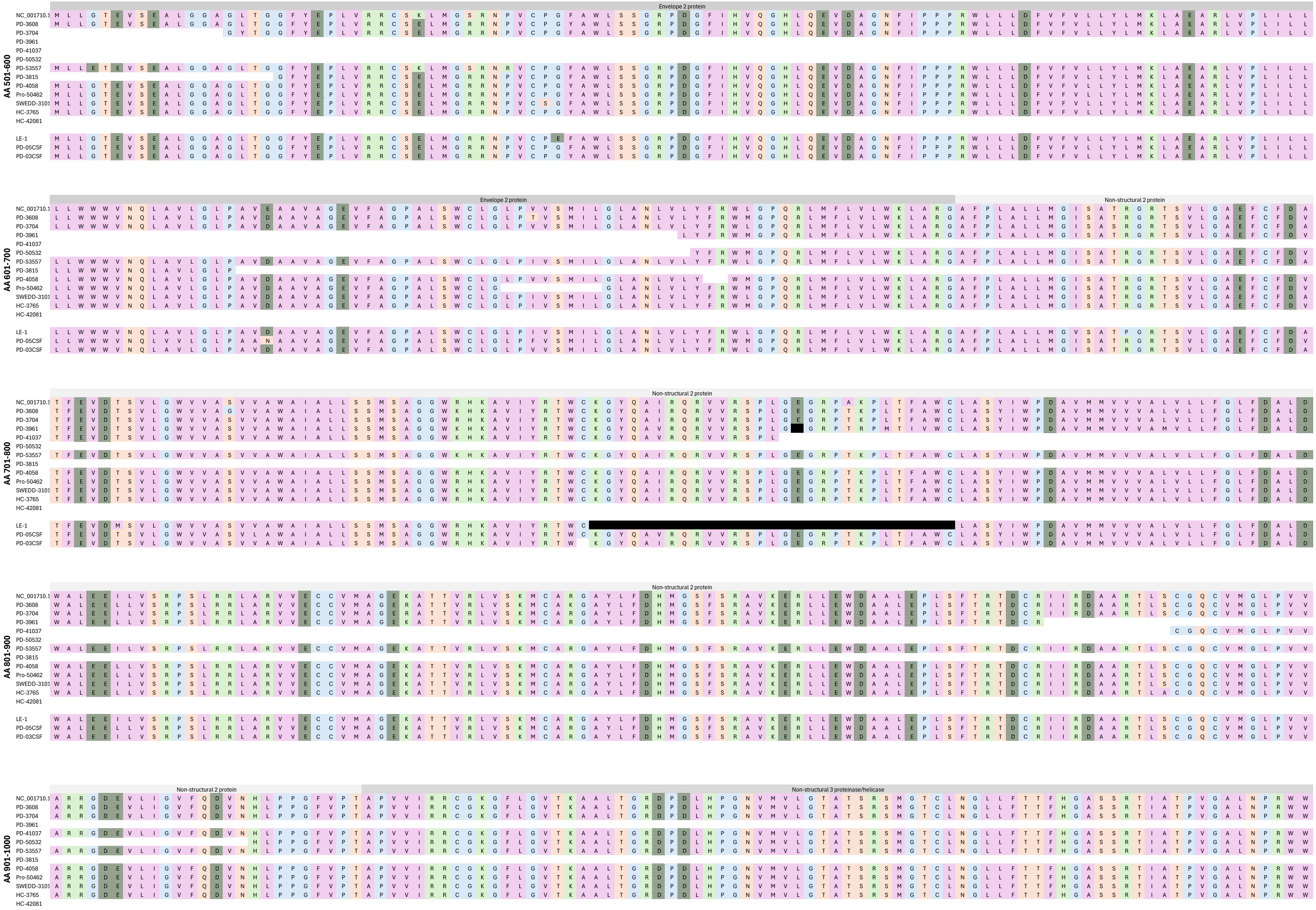

AA 501-600
Envelope 2 protein
AA 601-700
Envelope 2 protein
Non-structural 2 protein
AA 701-800
Non-structural 2 protein
AA 801-900
Non-structural 2 protein
AA 901-1000
Non-structural 2 protein
Non-structural 3 proteinase/helicase
NC_001710.1
PD-3608
PD-3704
PD-3961
PD-41037
PD-50532
PD-53557
PD-3815
PD-4058
Pro-50462
SWEDD-3101
HC-3765
HC-42081
LE-1
PD-05CSF
PD-03CSF

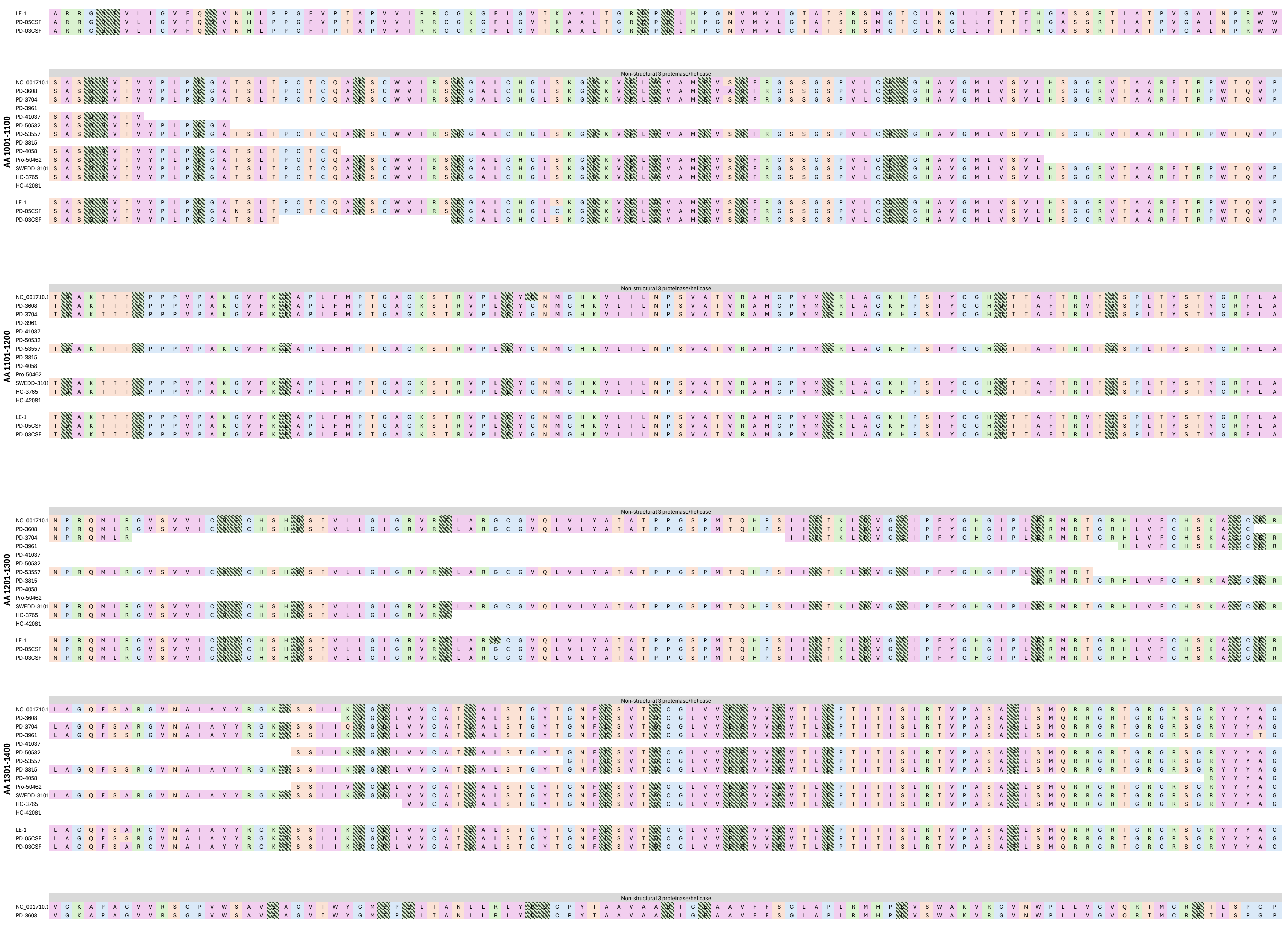
LE-1
PD-05CSF
PD-03CSF
Non-structural 3 proteinase/helicase
AA 1001-1100
AA 1101-1200
AA 1201-1300
AA 1301-1400
NC_001710.1
PD-3608
PD-3704
PD-3961
PD-41037
PD-50532
PD-53557
PD-3815
PD-4058
Pro-50462
SWEDD-3101
HC-3765
HC-42081

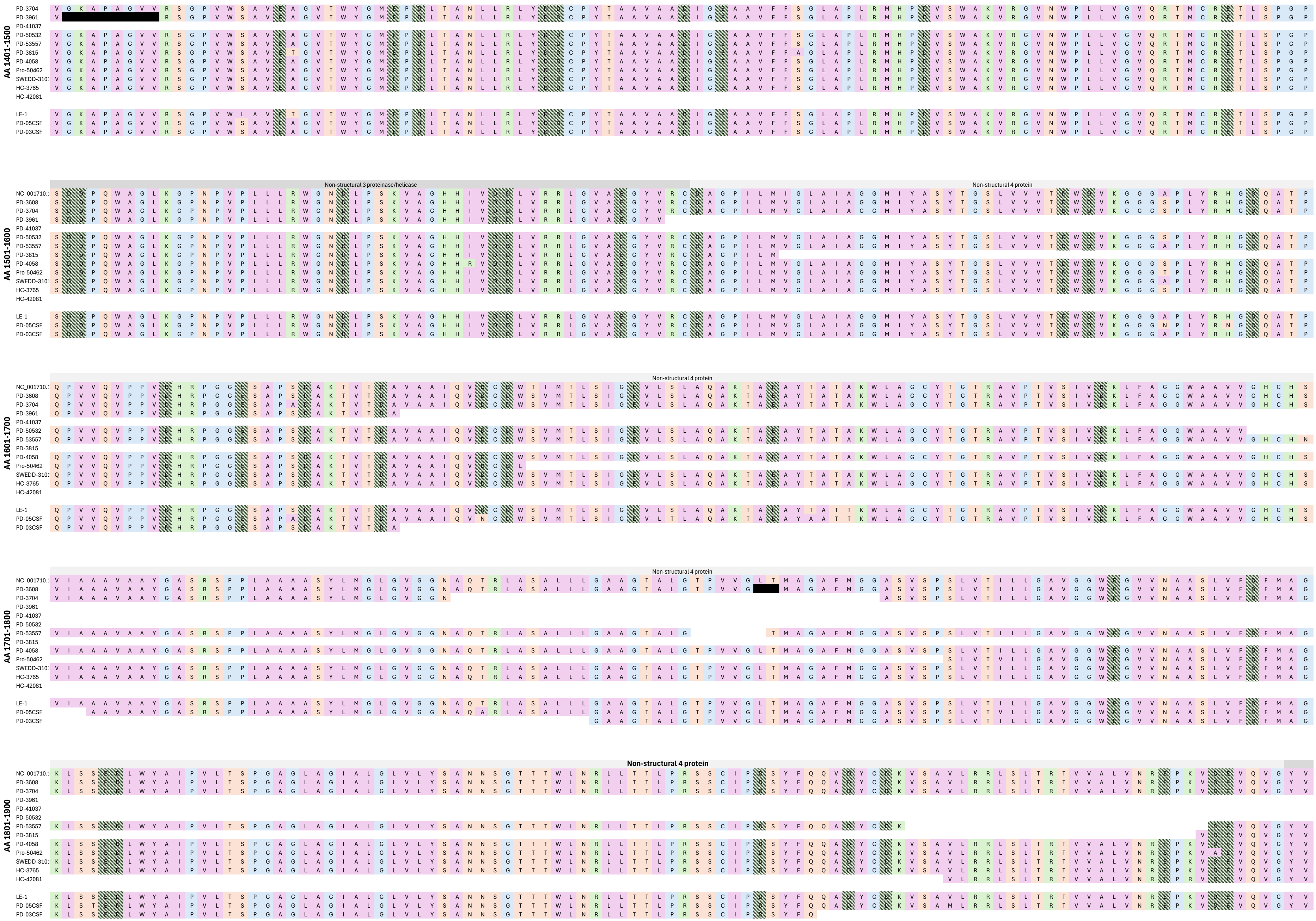

AA 1401-1500
AA 1501-1600
AA 1601-1700
AA 1701-1800
AA 1801-1900
Non-structural 3 proteinase/helicase
Non-structural 4 protein
NC_001710.1
PD-3608
PD-3704
PD-3961
PD-41037
PD-50532
PD-53557
PD-3815
PD-4058
Pro-50462
SWEDD-3101
HC-3765
HC-42081
LE-1
PD-05CSF
PD-03CSF

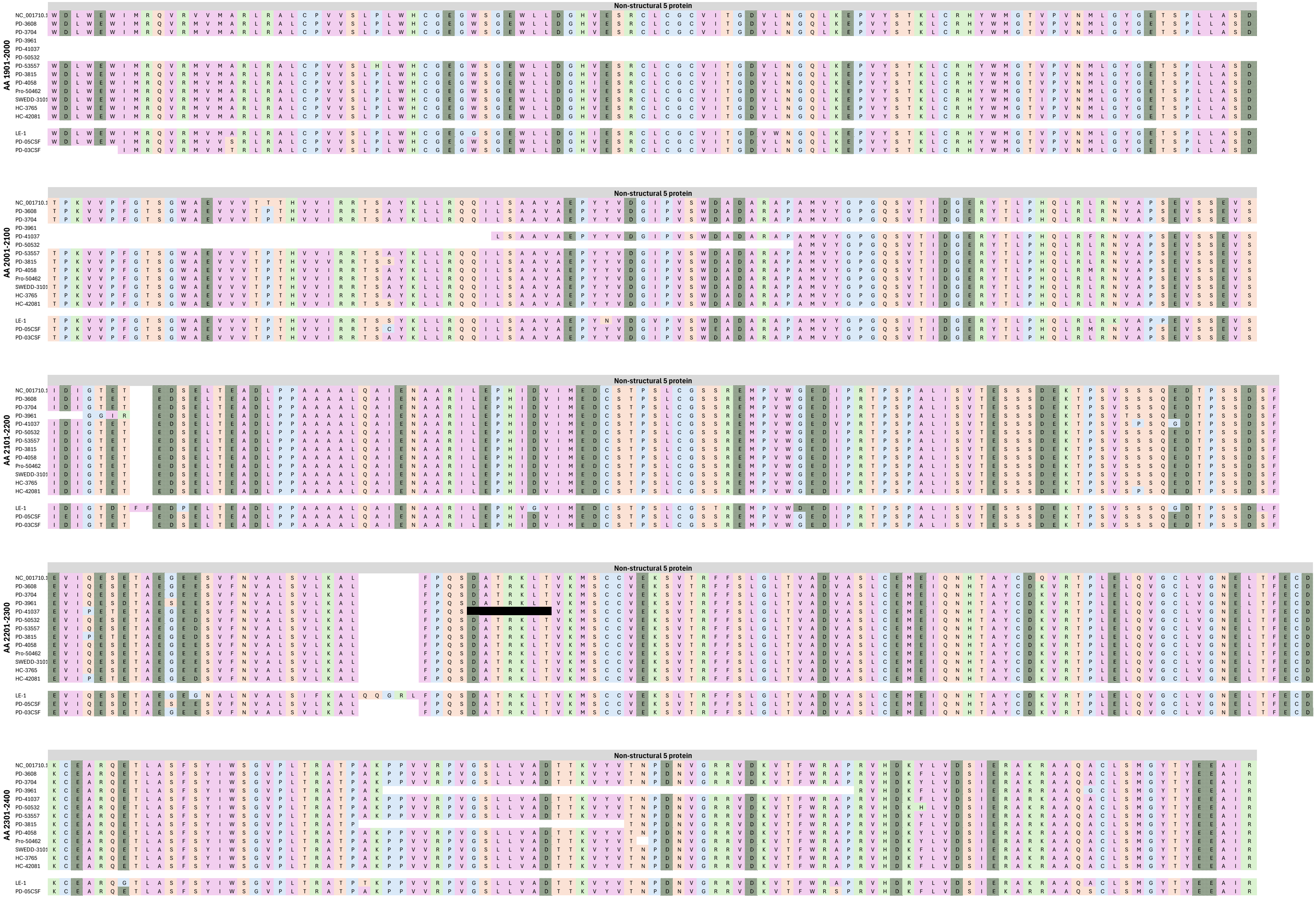

Non-structural 5 protein
AA 1901-2000
AA 2001-2100
AA 2101-2200
AA 2201-2300
AA 2301-2400
NC_001710.1
PD-3608
PD-3704
PD-3961
PD-41037
PD-50532
PD-53557
PD-3815
PD-4058
Pro-50462
SWEDD-3101
HC-3765
HC-42081
LE-1
PD-05CSF
PD-03CSF

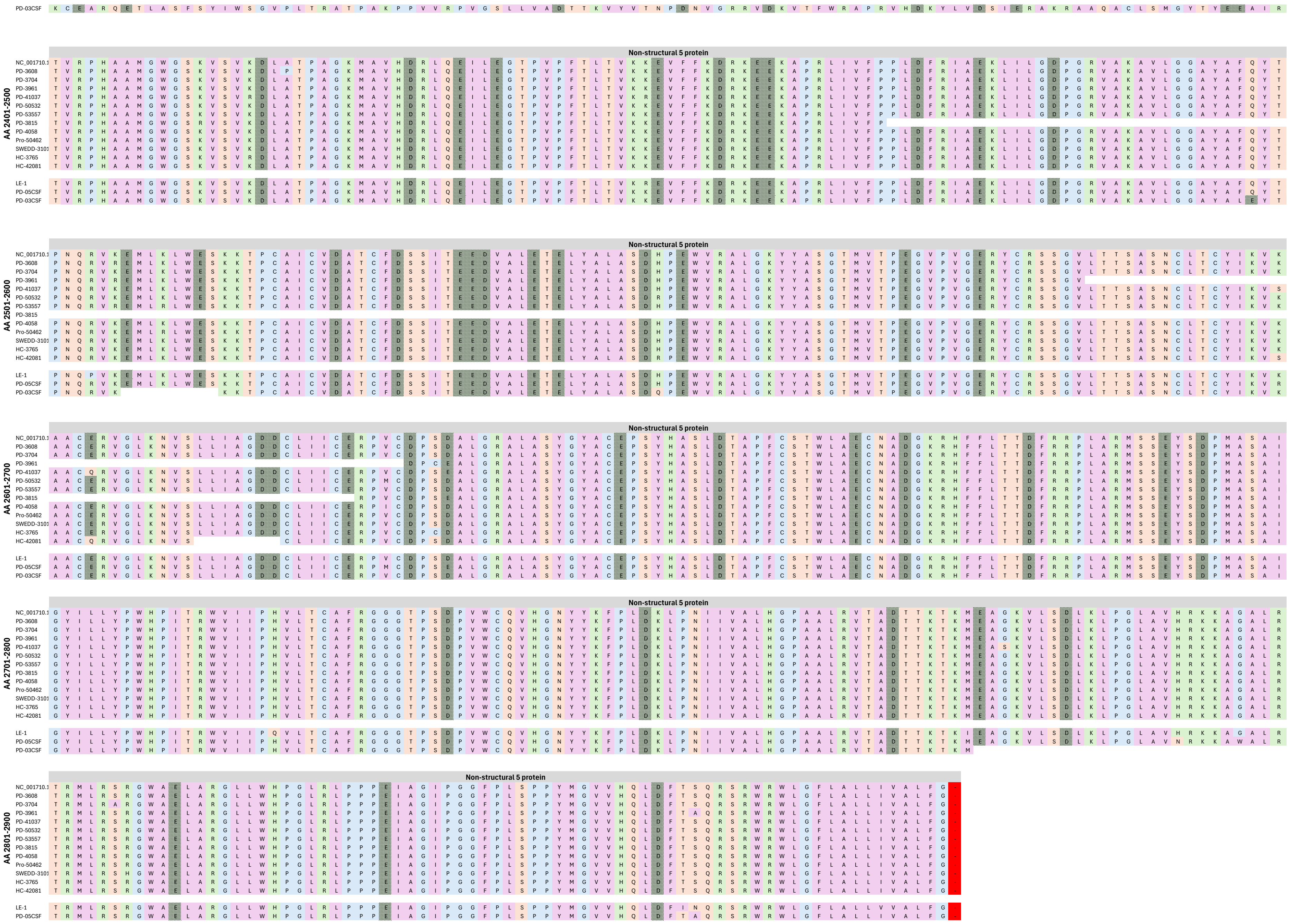

PD-03CSF
Non-structural 5 protein
AA 2401-2500
AA 2501-2600
AA 2601-2700
AA 2701-2800
AA 2801-2900
NC_001710.1
PD-3608
PD-3704
PD-3961
PD-41037
PD-50532
PD-53557
PD-3815
PD-4058
Pro-50462
SWEDD-3101
HC-3765
HC-42081
LE-1
PD-05CSF

Supplemental Figure 2

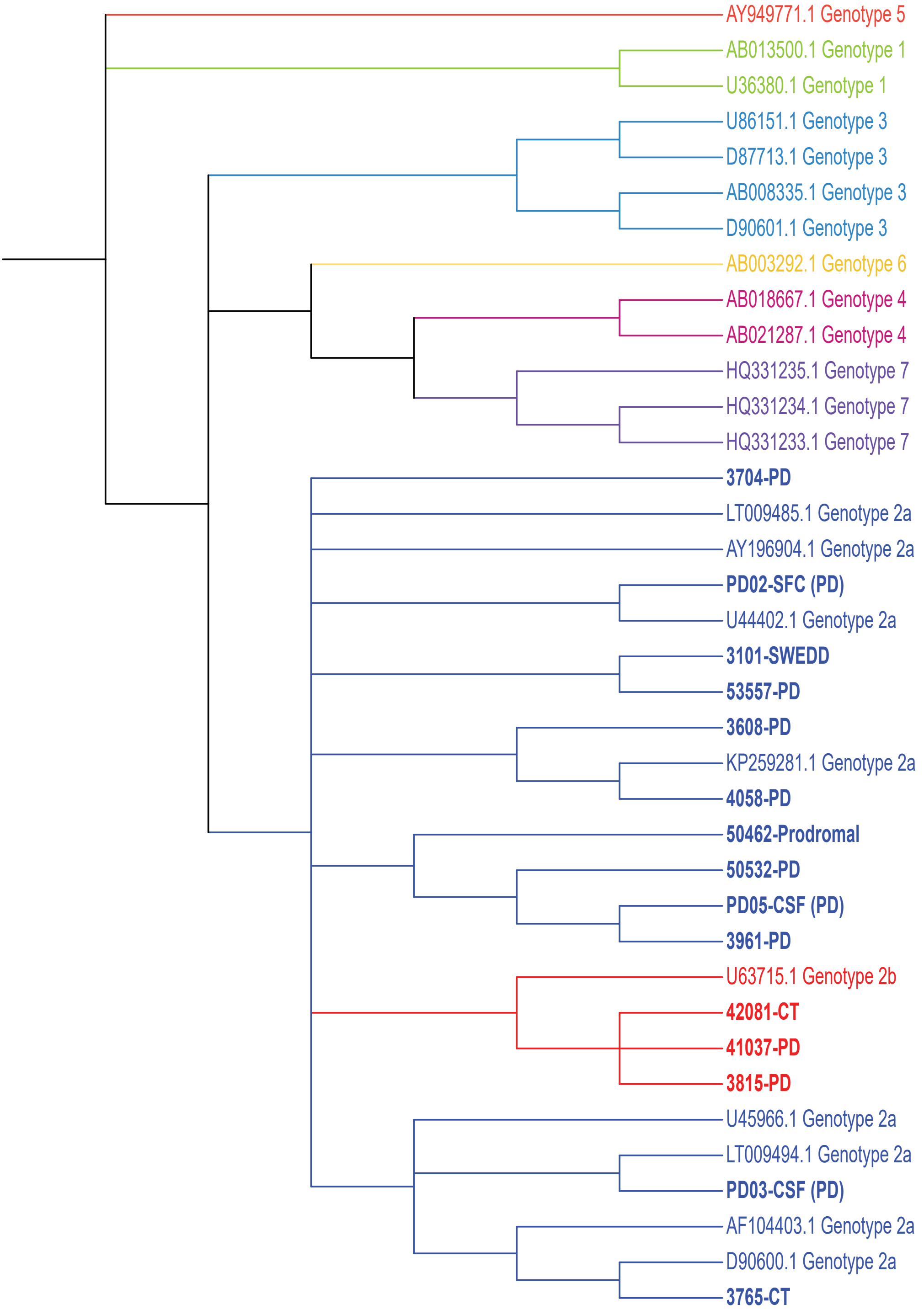

Supplement: Supplemental data [file jciinsight-10-189988-s257.pdf]
